# Identifying Lethal Dependencies with HUGE Predictive Power

**DOI:** 10.3390/cancers14133251

**Published:** 2022-07-01

**Authors:** Marian Gimeno, Edurne San José-Enériz, Angel Rubio, Leire Garate, Estíbaliz Miranda, Carlos Castilla, Xabier Agirre, Felipe Prosper, Fernando Carazo

**Affiliations:** 1 Departamento de Ingeniería Biomédica y Ciencias, TECNUN, Universidad de Navarra, 20009 San Sebastian, Spain; mgimenoc@unav.es (M.G.); arubio@tecnun.es (A.R.); ccastilla.1@tecnun.es (C.C.); 2 Programa Hemato-Oncología, Centro de Investigación Médica Aplicada, IDISNA, Universidad de Navarra, 31008 Pamplona, Spain; esanjose@alumni.unav.es (E.S.J.-E.); emelizalde@unav.es (E.M.); 3 Centro de Investigación Biomédica en Red de Cáncer (CIBERONC), 28029 Madrid, Spain; lgarate@unav.es; 4 Instituto de Ciencia de los Datos e Inteligencia Artificial (DATAI), Universidad de Navarra, 31080 Pamplona, Spain; 5 Departamento de Hematología, Clínica Universidad de Navarra, Universidad de Navarra, 31008 Pamplona, Spain

**Keywords:** *CRISPR*-Cas9 screening, precision medicine, synthetic lethality

## Abstract

**Simple Summary:**

This work shows that the predictions of lethal dependencies (LEDs) between genes can be dramatically improved by incorporating the “HUb effect in Genetic Essentiality” (HUGE) of gene alterations. In three genome-wide loss-of-function screens—Project Score, CERES score and DEMETER score—LEDs are identified with 75 times larger statistical power than using state-of-the-art methods. In AML, we identified LEDs not recalled by previous pipelines, including FLT3-mutant genotypes sensitive to FLT3 inhibitors. Interestingly, in-vitro validations confirm lethal de-pendencies of either NRAS or PTPN11 depending on the NRAS mutational status.

**Abstract:**

Recent functional genomic screens—such as *CRISPR*-Cas9 or RNAi screening—have fostered a new wave of targeted treatments based on the concept of synthetic lethality. These approaches identified LEthal Dependencies (LEDs) by estimating the effect of genetic events on cell viability. The multiple-hypothesis problem is related to a large number of gene knockouts limiting the statistical power of these studies. Here, we show that predictions of LEDs from functional screens can be dramatically improved by incorporating the “HUb effect in Genetic Essentiality” (HUGE) of gene alterations. We analyze three recent genome-wide loss-of-function screens—Project Score, CERES score and DEMETER score—identifying LEDs with 75 times larger statistical power than using state-of-the-art methods. Using acute myeloid leukemia, breast cancer, lung adenocarcinoma and colon adenocarcinoma as disease models, we validate that our predictions are enriched in a recent harmonized knowledge base of clinical interpretations of somatic genomic variants in cancer (AUROC > 0.87). Our approach is effective even in tumors with large genetic heterogeneity such as acute myeloid leukemia, where we identified LEDs not recalled by previous pipelines, including *FLT3*-mutant genotypes sensitive to *FLT3* inhibitors. Interestingly, in-vitro validations confirm lethal dependencies of either *NRAS* or *PTPN11* depending on the *NRAS* mutational status. HUGE will hopefully help discover novel genetic dependencies amenable for precision-targeted therapies in cancer. All the graphs showing lethal dependencies for the 19 tumor types analyzed can be visualized in an interactive tool.

## 1. Introduction

The traditional concept of synthetic lethality consists of the concurrent loss of functionality of two genes resulting in cellular death. A relevant example is the effectiveness of *PARP* inhibitors in tumors with inactivated *BRCA1* and *BRCA2* [1]. In recent years, the advances in functional genomics triggered by large-scale loss-of-function screening—such as *CRISPR*-Cas9 or RNA interference (RNAi) screens—have boosted the discovery of hundreds of novel targets and context-specific lethal dependencies (LEDs) [2,3,4,5,6,7], defined as any association between two genes that results in differential viability depending on their genetic context (Figure S1).

Several studies have carried out large-scale functional genomic screens to identify genome-wide targets and LEDs [2,3,4,5]. The Project Score [4], the Achilles Project [5,6] and the Project DRIVE [7] are three studies that performed genome-wide gene-knockouts in cancer cells aiming at establishing novel targets and LEDs. The refinement of computational and technical tools has improved the potential of loss-of-function screening to identify cancer vulnerabilities [3,8,9]. However, the multiple testing problem, related to a large number of gene knockouts, limits the statistical power of these studies and, therefore, their potential to find new targets.

Here, we show that previous efforts to predict LEDs from functional screening can be significantly improved by taking into account the “HUb effect” in Genetic Essentiality (HUGE) of some gene alterations: a few specific sets of gene alterations are statistically associated with large changes in the essentiality of multiple genes. These “hub” aberrations lead to more statistically reliable LEDs than other alterations that do not participate in such hubs. We incorporate the HUGE effect in the statistical analysis of three recent loss-of-function experiments of both The Project Score and The Achilles Project (two datasets) showing that the number of LEDs discovered for a given FDR considerably improves for both *CRISPR*-Cas9 and RNAi screens. 

Using acute myeloid leukemia (AML), breast cancer (BRCA), lung adenocarcinoma (LUAD) and colon adenocarcinoma (COAD) as disease models, we validate that the predictions are enriched in associations used in the clinic. Finally, we validated in vitro an example of a therapy guideline based on LED selection in AML. The HUGE analysis will help discover novel tumor vulnerabilities in specific genetic contexts, providing valuable candidates—targets and genetic variants as biomarkers—for further personalized treatments in hematological diseases or other cancer disorders.

## 2. Materials and Methods

### 2.1. Data Integration

Data of loss-of-function screens libraries (17,980 knockout genes in 412 cancer cell lines) of the project Achilles [10] were integrated with gene expression and their corresponding gene alteration profiles (gene variants in ~1600 genes) obtained from CCLE and Shao et al. [6]. We gathered gene expression of cells using RNA-seq data to confirm that the genes that were essential for a cohort of cells were expressed before the RNAi library experiment was performed [11]. Gene variant panels were filtered out using the parameters of CCLE’s authors to avoid common polymorphisms, low allelic fractions, putative neutral variants, and substitutions located outside of the coding sequence [12].

We used the DEMETER score [5,8] as a measure of gene essentiality of the RNAi libraries of the project Achilles [10]. DEMETER quantizes the competitive proliferation of the cell lines controlling the effect of off-target hybridizations of siRNAs by solving a complex optimization problem. The more negative the DEMETER score is, the more essential the gene is for a cell line. We imputed missing elements of DEMETER using the nearest neighbor averaging algorithm [13]. Moreover, we collected gene expression patterns from RNA-seq data [11] to confirm those essential genes are expressed when they are essential. Based on DEMETER data, we first identified genes that were essential for a selected tumor subtype. Essential genes were required to meet several criteria: (i) they must be essential for at least 20% of samples of the selected cancer subtype, (ii) they must be specific to the cancer type under study, i.e., they must be non-essential for other cancer types, and (iii) they must be expressed before RNAi experiment (>1TPM at least in 75% samples). 

### 2.2. Statistical Model

We developed a statistical algorithm to identify genes whose essentiality is highly associated with the genetic alteration of other genes. Dealing with this statistical issue implies solving a large multiple hypotheses problem (more than one million hypotheses). In similar scenarios, traditional corrections—such as Benjamini-Hochberg (BH), Bonferroni, or Holm—showed very few or no gene-biomarker LEDs for a given FDR [14]. To overcome this problem, we developed a covariate-based statistical approach—similar to the Independent Hypothesis Weighting procedure [14] (Figure S2). 

Let *e* denote the number of RNAi target genes and *n* denote the number of screened samples. Let **D** be an e×n matrix of essentiality whose entries $d_{ij}$ represent the DEMETER score for the RNAi target *i* in sample *j*. Let **m** be a m×n dichotomized matrix whose entry $m_{ij}$ denotes whether sample *j* is mutant or not according to the previous criteria:

(1) $$m_{ij}=\left\{\begin{array}{c} 1,\;if\;mutant\left(MUT\right)\\ 0,\;if\;wild-type\;\left(WT\right) \end{array}\right.\;,$$

Let **s** be a subset of n’ cell lines that yield an essentiality vector $d_{s}=\left(d_{e_{s}{}_{1}},\ldots ,\;d_{e_{s}{}_{n^{\prime }}}\right)$ for the *e*th RNAi target. Let $m_{s}=\left(m_{s_{1}}\ldots ,m_{s_{n}}\right)$ be the expression vector of a putative gene biomarker. The null hypotheses are defined as:

(2) $$H_{0}^{g}:\;E\left(d_{s}|m_{s}\in MUT\right)=E\left(d_{s}|m_{s}\in WT\right)$$

This null hypothesis is, therefore: “the expected essentiality of a gene knock-down is identical in mutant and wild-type cell lines”. To test this hypothesis, we used a moderated t-test implemented in limma [15]. We applied this test for each RNAi target and all the gene variants to obtain the corresponding *p*-values (Figure S2). Dealing with these *p*-values implies correcting for multiple hypotheses. 

In our case, we divided the *p*-values corresponding to all the tests into n groups, where n is the number of altered genes. For each of these groups, we computed the local false discovery rate (local FDR) [16]. The local FDR estimates, for each test, the probability of the null hypothesis to be true, conditioned on the observed *p*-values. The formula of the local FDR is the following:

(3) $$P\left(H_{0}|z\right)=localFDR\left(z\right)=\frac{\pi_{0}f_{0}\left(z\right)}{f\left(z\right)}\;,$$

where *z* is the observed *p*-values, π_0_ is the proportion of true null hypotheses—estimated from the data, $f_{0}\left(z\right)$ the empirical null distribution—usually a uniform (0, 1) distribution for well-designed tests—and $f\left(z\right)$ the mixture of the densities of the null and alternative hypotheses, which is also estimated from the data.

As stated by B. Efron and R. Tibshirani [16], “the advantage of the local FDR is its specificity: it provides a measure of belief in gene i’s ‘significance’ that depends on its *p*-value, not on its inclusion in a larger set of possible values” as it occurs, for example, with *q*-values or the standard FDR. The local FDR and π_0_ were estimated using the Bioconductor’s R Package *q*-value [17]. 

### 2.3. Comparison with the Project Score

To compare our results with Project Score’s ones, we selected the same 12 primary cancer tissues shared in both datasets. The comparison followed two steps: (1) using CCLE and DEMETER scores with the Project Score’s algorithm, (2) running our approach adapted to Project Score conditions. In the first step, following the code published in their work, an ANOVA test was performed on each tissue to calculate all possible dependent partners. The Storey–Tibshirani correction was then used, using the criteria mentioned in Project Score methods [4]. This enabled us to correct the ANOVA *p*-values and obtain significant associations. Secondly, the comparison between both methodologies was only possible if the same adjusted *p*-value is calculated for both datasets. Therefore, we estimated the FDR with our data as it is the *q*-value selected by the Project Score. The FDR correction was obtained using the Bioconductor R package *IHW* [14], which enables the consideration of covariates-based multiple hypothesis correction, as well as estimating the FDR. Discoveries from both methodologies in DEMETER and CCLE datasets were plotted in different volcano plots, and the number of significant LEDs was counted (FDR < 20%).

### 2.4. Integration of the VICC Knowledgebase of Clinical Interpretations of Genomic Variants

We downloaded 19,551 clinical interpretations of somatic genomic variants in cancer from the Variant Interpretation for Cancer Consortium (VICC) [18,19] (version December 2020). We filtered out incomplete (e.g., entrees without annotated drug or biomarker) and redundant associations. We then selected all associations that are annotated with acute myeloid leukemia (AML) and synonyms. From all drugs, we selected those that have an annotated protein target. To do so, we retrieved the data publicly available in the ChEMBL [20] and DrugBank [21] online repositories. In total, 216 out of 19,551 associations matched these criteria. We consider a true positive if either HUGE or ST identifies an LED whose mutation biomarker coincides with a VICC’s association and the protein target is included in the same association, or at least in a gene of the same pathway in the STRING database (v.11, STRING score threshold = 400; default value on STRING for “medium” confidence) [22].

We calculated ROC and PR curves considering the two top evidence levels included in VICC [18,19], namely, (i) evidence from professional guidelines or FDA-approved therapies; and (ii) evidence from clinical trials or other well-powered studies in clinical populations, with expert consensus.

### 2.5. Application to Acute Myeloid Leukemia (AML) as a Disease Model 

We applied the pipeline to the AML cohort of cell lines (*n* = 15). In the first step, essential genes were required to be: (i) essential for at least 25% AML samples, (ii) specific for AML cells, and (iii) expressed before the RNAi experiment. The algorithm outputs a ranking of significant gene pairs (LEDs) that consist of a couple of genes in which the first one is essential depending on the genetic alteration of the other.

For the final ranking for AML, we selected those LEDs that showed a *p*-value < 0.05 and local FDR ≤ 0.6, |D DEMETER| > 2 (default value suggested by DEMETER’s authors). Additionally, we interrogated which of these LEDs had direct relationships (co-expressed, annotated in the same pathway database, or contained in a common experiment) in the STRING database [22] to ensure there is an established biological relationship between the essential gene and the subrogate biomarker. This biological double-check is not necessary and can be omitted when the researcher looks for novel relationships. 

In vitro validation was performed using siRNAs against *NRAS* and *PTPN11* in four different AML cell lines, two with *NRAS*-genetic variants (HL-60 and OCI-AML3) and two *NRAS*-wt cell lines (MV4-11 and HEL). Finally, the model was compared with 3 standard statistical methods (namely Benjamini-Hochberg (BH), Bonferroni and Holm) known to have suboptimal sensitivity (recall of true positives) in specific scenarios in 19 additional tumor subtypes to define the potential for controlling the FDR [14]. See File S1 for more details. 

## 3. Results

### 3.1. Gene Variants Associated with Multiple Essential Genes Increase the Power of Loss-of-Function Screens

One of the main statistical challenges to finding LEDs by integrating genome-wide functional screens with -omics datasets is the multiple hypothesis testing problem. Correction for multiple hypotheses reduces the statistical significance of results (meaning a decreased detection rate and an increased false-positive rate). The Project Score presented a large-scale genome-wide *CRISPR*-Cas9 screening analysis targeting 18,009 genes in 30 different cancer types, across 14 different tissues [4,23]. They presented a methodology to detect LEDs based on finding differences in genetic essentiality in cell lines associated with the presence of specific gene variants (ANOVA test [24] with the Storey–Tibshirani *p*-value correction). Following this procedure, the Project Score was able to identify genetic LEDs in 7 out of 14 individual tissues analyzed [4,23].

**Figure 1:**
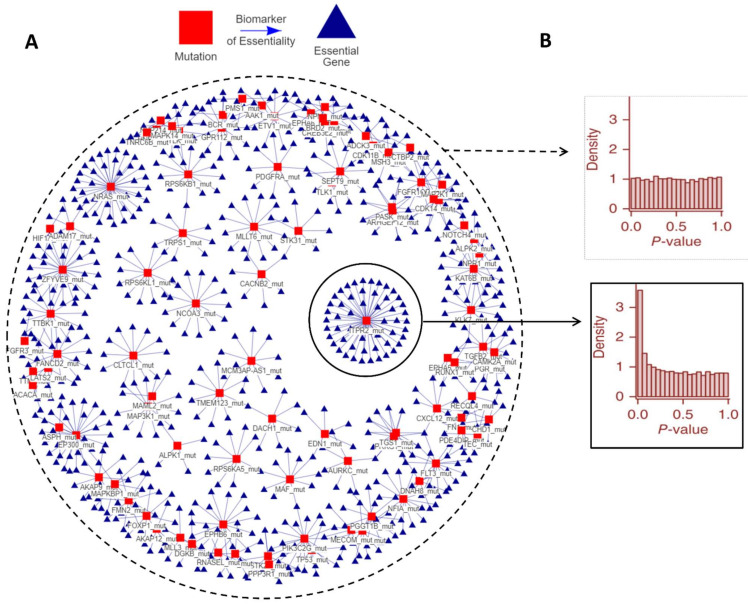
**The hub effect in genetic essentiality in Acute Myeloid Leukemia.** In each cell, a small set of gene aberrations is associated with large changes in genetic essentiality. (**A**) A bipartite graph in which red squares represent gene variants (e.g., mutations), blue triangles represent significant changes in cell viability related to knocked-down genes. Both nodes are linked by a line if the variations in the essentiality have a statistically significant association with the presence of the gene variant. (**B**) Implications in *p*-value histograms of the HUGE effect. Hub associations show a high peak close to zero *p*-values indicating that the null hypothesis is rejected in more cases and that these genetic variants are associated with a higher response to the inhibition of more gene products. Segregating the statistical analysis according to the alteration provides more statistical power. Essential genes and other tumor types can be visualized in https://fcarazo.shinyapps.io/visnetShiny/ (accessed on 24 June 2022). Abbreviations. HUGE: The hub effect in genetic essentiality.

Analyzing Project Score’s data, we observed that for each tumor type, a few specific genetic alterations were significantly associated with the genetic essentiality of a large set of genes. This handful of genetic aberrations shows a hub effect, in which a gene variant is associated with large changes in the essentiality of multiple genes. We termed this behavior the “HUb effect in Genetic Essentiality” (HUGE) (Figure 1A; other tumor types can be visualized in https://fcarazo.shinyapps.io/visnetShiny/ (accessed on 24 June 2022)). From the point of view of statistics, the HUGE effect is defined as an improvement of the statistical power by using gene variants as co-variates in a multiple hypothesis problem. Other biological covariates such as gene expression or copy number alterations have also shown to be covariates that increase the statistical power [14]. Using gene variants as statistical covariates provides a larger number of positives for a given FDR, which consequently means an increased specificity and sensitivity, or type I and type II errors, as demonstrated in File S1, Section S6. Interestingly, the analysis shows that the HUGE effect is present in all tumors analyzed, significantly improving the predictive power of LEDs. 

The presence of the HUGE effect in a cancer type can be also understood as a predictive model in which each mutation has a different capability to define the genetic essentiality of multiple genes. To show it visually, the histogram of *p*-values of a gene alteration represents how gene alterations are associated with the genetic essentiality of multiple genes. Histograms of the *p*-values for alterations that conform to a “hub” show a peak near the origin, which means that cells with these alterations are sensitive to the depletion of a large number of genes (Figure 1B). Conversely, if the hubs of alterations are not considered, the relationships of mutations and viability show a flat histogram of *p*-values. This does not necessarily mean that such relationships are not biologically relevant, but that it is difficult to distinguish them from random associations and will be considered as artifacts after multiple testing corrections.

The HUGE effect helps palliate the multiple hypothesis correction problem. Using the mutation under study as a covariate, multiple hypotheses can be differently treated considering the overall association of gene alteration in the complete set of essential genes (Figures S2 and S3). Using this concept, we developed a statistical model that integrates HUGE information to find LEDs (Figure S2). 

**Figure 2:**
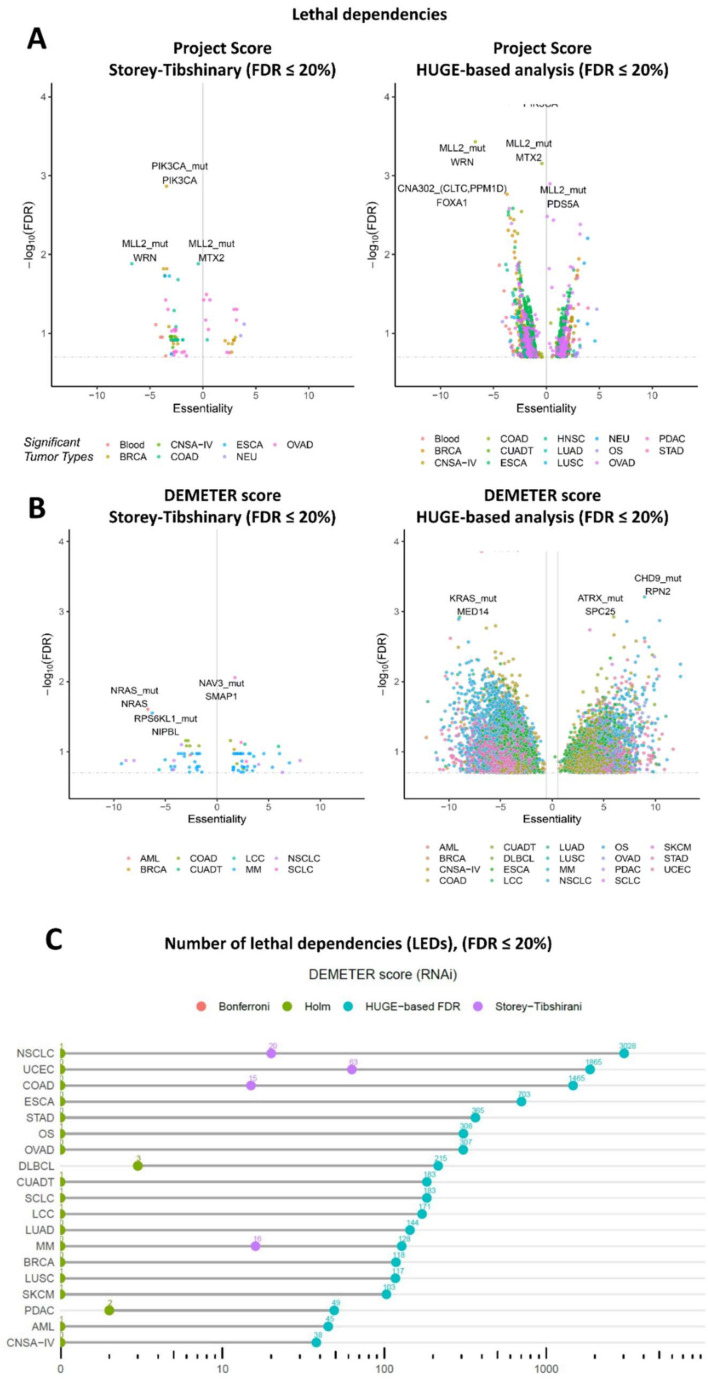
**HUGE-based analysis with Project Score and Achilles Project datasets.** (**A**) Volcano plots of lethal dependencies, LEDs, identified in the Project Score dataset. From left to right: (i) result of Project Score, (ii) results of analyzing Project Score dataset with the HUGE-based methodology. Each dot represents a significant LED (FDR < 20%). The X-axis represents the difference in gene essentiality when the event (gene variants) is present. The Y-axis represents the FDR values (−log10) for that change. (**B**) Equivalent volcano plots using Achilles Project. From left to right: (i) results of Achilles Project analyzed with the standard procedure, (ii) results of analyzing Achilles Project dataset with HUGE-based methodology. (**C**) The number of LEDs found (FDR ≤ 20%) in 19 tumors of the DEMETER score (RNAi) and 22 tumors of the CERES score (*CRISPR*-Cas9) using standard statistical pipelines (Storey–Tibshirani, Bonferroni, and Holm) and the HUGE-based algorithm. Bonferroni and Holm return the same number of hypotheses in all cases. Abbreviations. LED: lethal dependency; ALL: acute lymphoblastic leukemia; AML: acute myeloid leukemia; BRCA: breast ductal carcinoma; CNSA-IV: central nervous system astrocytoma grade IV; COAD: colon adenocarcinoma; CUADT: upper aero-digestive tract squamous cell carcinoma; DLBCL: diffuse large B-cell lymphoma; ESCA: esophagus squamous cell carcinoma; KIRC: kidney renal clear cell carcinoma; LCC: lung large cell carcinoma; LUAD: lung adenocarcinoma; LUSC: lung squamous cell carcinoma; MM: multiple myeloma; NSCLC: non-small cell lung carcinoma; OS: osteosarcoma; OVAD: ovary adenocarcinoma; PDAC: pancreas ductal carcinoma; SCLC: small cell lung carcinoma; SKCM: skin carcinoma; UCEC: endometrium adenocarcinoma.

Previous efforts to correct multiple testing in this problem consider a single set of tests (all gene aberrations and *CRISPR*-Cas9 knockouts) and apply a correction that controls the FDR, such as Storey–Tibshirani (ST), as performed in the Project Score. Interestingly, in all tumors, our approach increases the statistical power of the analysis. From a statistical point of view, a flat histogram is compatible with the null hypothesis for all the tests and, therefore, multiple hypothesis correction drives to none or few discoveries (Figure S4). Every single tumor shows *p*-value histograms related to specific gene variants that have a higher zero-peak than the histogram associated with all tests in such tumor (Figures S5–S23). To test this approach, we compare the results using HUGE with previous LED identification strategies in three genome-wide functional genomic projects: The Project Score [4], the DEMETER score and the CERES score (DEMETER and CERES are included in the Achilles Project [5,6]). First, to test the potential of HUGE to predict LEDs with *CRISPR*-Cas9 screens, we analyze the Project Score dataset [4]. Project Score integrates 215 different genetic events across 14 tumor types, including SNVs and CNVs. In the same reference, the authors found at least one LED in 7 out of the 14 tumor types analyzed. A total of 40 out of 215 events were detected to be significant biomarkers of essentiality (FDR ≤ 20%), which correspond to 77 unique LEDs (a single genetic event can be associated with several essential genes). Analyzing Project Score’s data using the HUGE-based methodology, we identify 1438 unique associations with the same FDR (18 times larger than Project Score, Figure 2A), corresponding to 80 single genetic events. Moreover, using HUGE we detect at least one LED in all the 14 tumors analyzed, finding LEDs in 10 tumors that would have been missed using the original pipeline, affecting around 10–20 genes for each disease type. 

We also tested HUGE in the DEMETER score of the Achilles Project to predict LEDs, in this case using RNAi screening. The DEMETER dataset [5,10] is a large-scale genome-wide experiment of RNA interference libraries (17,085 knockdown genes) in 19 tumor types (Table S5). We integrate the DEMETER data with the corresponding cell line gene alteration profiles (genetic variants in ~1600 genes) obtained from the Cancer Cell Line Encyclopedia (CCLE) [12] and Shao et al. [6]. This integration turns out to have 27 Million hypotheses, which will hardly impair *p*-values after multiple hypothesis correction (Figure S2). Then, we replicate the Project Score’s pipeline with the DEMETER dataset and compare it with the HUGE-based approach to find LEDs, also including in the comparison other two standard *p*-value corrections used to control the FDR, namely Holm and Bonferroni. Using the standard ST procedure, we find 126 LEDs (FDR ≤ 20%). There are LEDs for 7 out of 19 tumors. The same dataset and FDR threshold using the HUGE-based approach provides 9535 LEDs (75.7 times larger than using ST). All cancer types (19 out of 19) showed significant LEDs in the HUGE-based analysis (Figure 2B). HUGE identifies 1,675 LEDs in six tumor types in which other methods recall no LEDs (FDR ≤ 20%); and 9409 LEDs in 19 tumor types that would have been missed using previous procedures (FDR ≤ 20%; Figure 2C). These results show that the HUGE effect is present with different intensities in all tumor types analyzed (Figures S5–S23).

As a further test of the increased predictive power of HUGE, we carry out a similar analysis using the CERES score, a *CRISPR*-Cas9 experiment of 22 tumors also included in the Achilles Project. In this case, the number of significant pairs is enriched 14 times over the standard approaches (FDR ≤ 20%; Figure S24).

### 3.2. LEDs Predicted by HUGE Have Better Validation Rates Than Standard Approaches

Validating a ranking of LEDs is not a simple task: it is desirable to have a gold standard of a disease-specific list of validated target-biomarker associations. We select as our gold standard The Variant Interpretation for Cancer Consortium (VICC) Meta-Knowledgebase [18,19]. This database integrates different datasets of clinical associations and includes the level of evidence for each entry: spanning from professional FDA guidelines to preclinical findings.

We test the enrichment in associations included in VICC in four tumor types, namely acute myeloid leukemia (AML), breast cancer (BRCA), lung adenocarcinoma (LUAD) and colon adenocarcinoma (COAD) for both HUGE and standard statistical methods. The VICC knowledgebase integrates (in September 2021) 19,551 clinical interpretations of somatic genomic variants in cancer of both resistant and sensitive biomarkers. We delete duplicated and incomplete associations, focused on those related to confirmed mutations and manually selected associations that match each tumor type (including synonyms).

We first run the two procedures (HUGE and Storey-Tibshirani; ST) with AML cell lines (Table S5) to find LEDs and compare how many LEDs predicted by HUGE and by ST are included in the VICC knowledgebase. For instance, if HUGE or the ST procedure predicts *FLT3* mutant AML genotypes to be sensitive to *FLT3* inhibition, it will be considered a true positive LED, as *FLT3* is a well-known target of AML and mutations in *FLT3,* the fms-like receptor-type tyrosine-protein kinase [25,26], are known to be sensitive biomarkers of the effectiveness of most *FLT3*-inhibitors [27,28]. 

**Figure 3:**
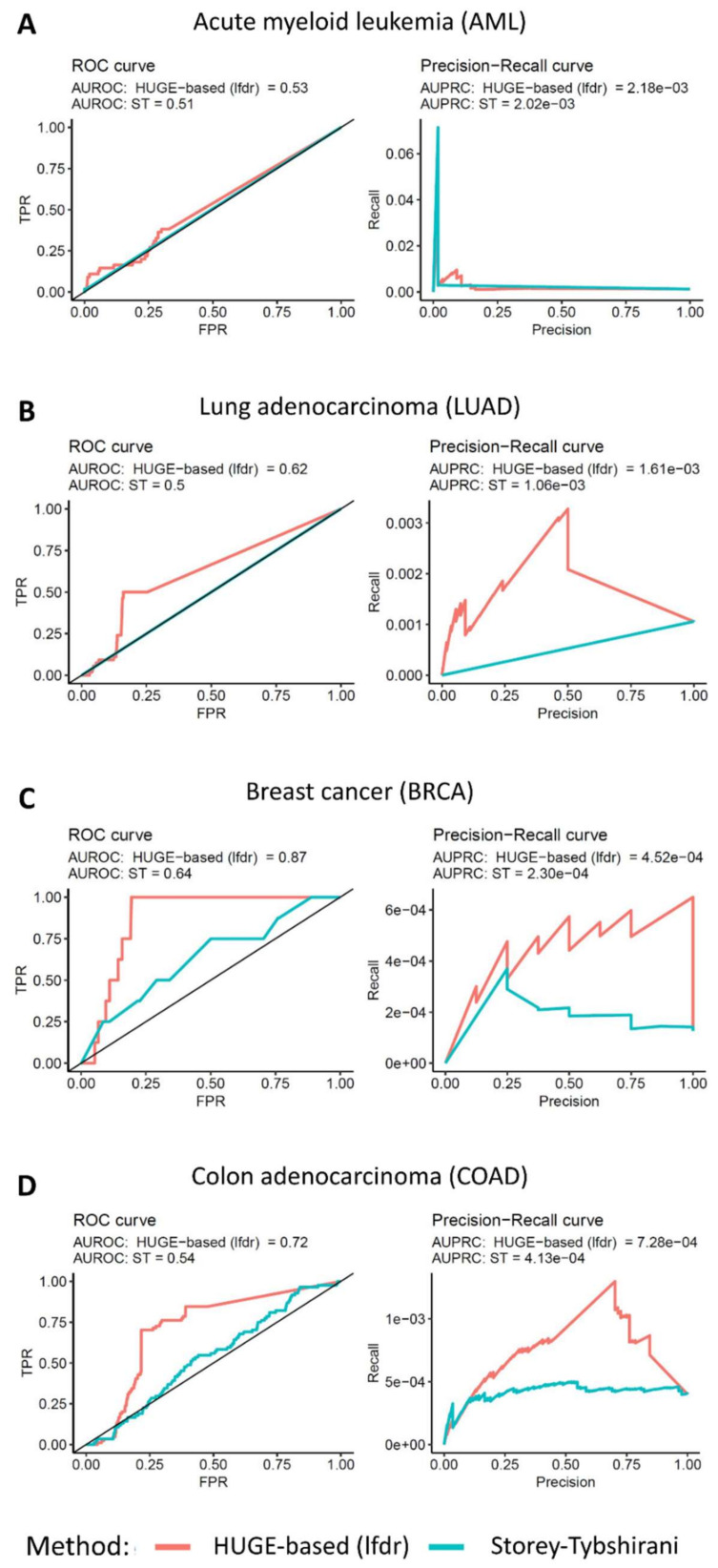
**ROC and precision-recall curves of four tumor types.** (**A**) Acute myeloid leukemia, (**B**) lung adenocarcinoma, (**C**) breast cancer and (**D**) colon adenocarcinoma. True positives were extracted from the knowledge base of the Variant Interpretation for Cancer Consortium [18,19]. For each tumor type, we selected only those associations that belong to the three highest levels of confidence (Level A: Evidence from professional guidelines or FDA-approved therapies relating to a biomarker and disease; Level B: Evidence from clinical trials or other well-powered studies in clinical populations, with expert consensus; and Level C: Evidence for therapeutic predictive markers from case studies, or other biomarkers from several small studies, or evidence for biomarker therapeutic predictions for established drugs for different indications).

In total, 216 out of 19,551 associations matched these filters. Getting the top 500 LEDs according to the ranking using the HUGE algorithm with AML, we find 17 LEDs that match the VICC knowledgebase of known clinic relationships (Table S1; Fisher *p*-value < 1 × 10^−^^51^). An equivalent analysis using the standard pipeline (ANOVA test [24] with the Storey–Tibshirani *p*-value correction) shows that out of the top 500 LEDs, only one is included in the VICC knowledgebase (Table S1; Fisher *p*-value = 6.551 × 10^−^^3^). This means that HUGE analysis identifies 16 true positive dependencies not recovered by ST (Fisher *p*-value = 6.41 × 10^−^^5^). The global value of AUROC (0.53) is not too far from the baseline of 0.5 (Figure 3A), perhaps because of the scarcity of true positives in our gold standard. We perform the same analysis with LUAD, BRCA and COAD getting AUCROC values of 0.62 (vs. 0.5), 0.87 (vs. 0.64) and 0.72 (vs. 0.54) for HUGE and ST, respectively. All cases show better values for HUGE than for ST (Figure 3B–D and Figure S25). 

### 3.3. Applying HUGE Methodology to Acute Myeloid Leukemia Cell-Lines Discovers Potential Therapy Biomarkers

AML is a hematologic neoplasm characterized by a remarkable phenotypic and genomic heterogeneity [29], a challenging disease model to test the applicability and impact of HUGE. We run the complete HUGE pipeline with AML and validate in vitro two of the predicted LEDs. 

As a preliminary step, we identify the potential genes that are essential for AML cell survival. The Achilles Project yielded 443 essential genes that are essential and specific for AML cells compared to other tumors (Table S2). Some of these genes belong to pathways known to be deregulated in AML (e.g., *MYB* [30] or *CEBPA* [31]). Interestingly, 160 of these 443 genes have previously been identified as potential cancer drivers in hematological malignancies according to the Candidate Cancer Gene Database (*p*-value = 7.76 × 10^−^^5^, Fisher exact test) [32].

**Figure 4:**
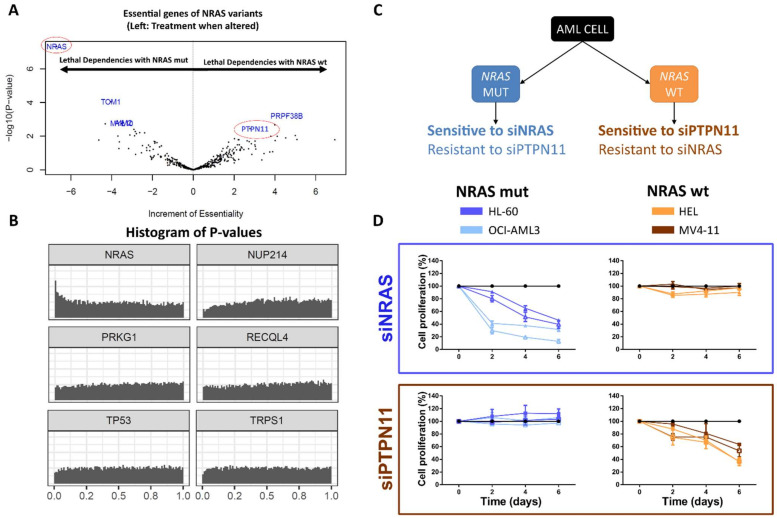
**Gene variants-based treatment guidelines in acute myeloid leukemia.** (**A**) Volcano-plot of lethal dependencies, LEDs, related to NRAS genetic mutations (left; MUT) and wildtype (right; WT) phenotypes. Increment of Essentiality and −log10 (*p*-value) are shown on X-axis and Y-axis, respectively. (**B**) Histogram of *p*-values for 6 genetic sequence variants in acute myeloid leukemia. NRAS-alteration is enriched in close to zero *p*-values, which is the basic concept of HUGE-based statistical approach. All genetic variants histograms of *p*-values can be found in the Supplementary Material. (**C**) Summary of the computational predictions validated: NRAS-altered cells were predicted to be sensitive to siNRAS and resistant to siPTPN11. Conversely, NRAS-wt cells were predicted to be sensitive to siPTPN11 and resistant to siNRAS. (**D**) Tumor proliferation of the four AML cell lines after inhibiting NRAS (siNRAS) and PTPN11 (siPTPN11) with specific siRNAs. Blue: NRAS-altered AML cell lines (HL-60 and OCI-AML3); Orange: NRAS-wild-type AML cell lines (MV4-11 and HEL).

We then run the HUGE algorithm to identify genomic alterations that could be defined as LED partners of those 443 essential genes. In this pipeline, we require predicted pairs to be biologically related to each other in the STRING database (see Online Methods). LED associations can be broken down into three groups regarding their dependency type: *positive lethal dependency (pLED)*, when a gene variant marks sensitivity to the inhibition of another gene; *negative lethal dependency (nLED)*, when a gene variant marks resistance to the inhibition of another gene; or *dual lethal dependency (dLED)*, when the same gene variant confers, concurrently, sensitivity to the inhibition of one gene and resistance to the inhibition of another gene (Figure S1). In total, we predict 24 LEDs, (12 *pLEDs* and 12 *nLEDs*, including two *dLEDs*; *p*-value < 0.05, local FDR ≤ 0.6 and |ΔEssentiality| > 2; Figure 4A, Table 1, Figure S26, and Table S3). Using the standard multiple hypotheses correction only one dependency turns out to be statistically significant. We provide the identified LEDs for the 19 tumors included in the Achilles Project following a similar pipeline (Tables S6–S24).

*NRAS* mutation ranks first in the analysis. Lethally dependent partners associated with *NRAS* genetic sequence variants show a *p*-value histogram that peaks at the origin (Figure 4A,B), meaning that *NRAS* mutations are associated with more tumor vulnerabilities than other alterations. Interestingly, *NRAS* alteration forms a *Dual Lethal Dependency* with *PTPN11* (Table 1, Figure 4C): it confers tumor sensitivity to *NRAS* inhibition and resistance to *PTPN11* inhibition. 

To validate our prediction, we first check that both *NRAS* and *PTPN11* siRNAs efficiently decreased the *NRAS* and *PTPN11* expression, respectively, in four AML cell lines (Figure S27). Then, we confirm the computational hypothesis: the downregulation of *NRAS* significantly decreases cell proliferation only in the *NRAS*-altered AML cell lines, and the inhibition of *PTPN11* expression produces an equivalent effect, specifically in the *NRAS*-wt AML cell lines (Figure 4D), validating the predicted dLED. Remarkably, the validated *PTPN11-NRAS*-wt pair was not detected using standard methodologies.

## 4. Discussion

The advent of large-scale functional genomic screens has allowed the identification of hundreds of novel gene targets and the prediction of genome-wide LEDs [4,33]. This strategy has multiplied treatment strategies, as using LEDs, the drug targets can be decoupled from their corresponding predictive biomarkers. The main statistical limit to finding LEDs is the large number of hypotheses that result from integrating gene essentiality and genetic functional events. In this work, we present HUGE, a novel analysis of *CRISPR*-Cas9 and RNAi large-scale screens that significantly improves the predictive power to find LEDs from loss-of-function screens in human tumors. It relies on the fact that some gene alterations are statistically related to the essentiality of large sets of genes. Using this characteristic as a prior covariate we significantly improve the predictive power of LEDs. 

Notably, the presence of the HUGE effect does not necessarily mean biological causality. HUGE dependencies are more statistically reliable than others, but this does not imply that predicted alterations are the major players in tumor development thus, they are not necessarily driver genes, i.e., they are just genetic biomarkers of gene essentiality. In other words, the Hub-Effect is a statistical association. Since “correlation does not imply causation” is not legitimate to deduce a cause-and-effect relationship between the presence of a mutation and the sensitivity to knocking down a gene. Even more, it cannot be concluded that the HUGE top-ranked genes (either the mutations or the knockdown genes) are driver genes. This would require further experimentation and validation. HUGE simply computes biomarkers of the vulnerability to a knockdown gene, that in turn, could be targeted by a drug. However, the fact that gene alterations co-occur with multiple LEDs in genetic hubs can be exploited to improve the statistical power.

To measure the increased predictive power of HUGE, we carry out three different comparisons within three functional genomic datasets: the Project Score, the DEMETER score and the CERES score. HUGE identifies LEDs with 14 and 75 times larger statistical power than using state-of-the-art methods in *CRISPR*-Cas9 and RNAi, respectively. However, it could be argued that this result could be an artifact of the statistical technique and that lowering the threshold for standard procedures would provide LEDs with similar reliability. This is not the case. As shown in the results, using the same number of predictions, HUGE’s results are more enriched in clinically used biomarkers than ST’s results. Remarkably, 1 of the 16 LEDs only identified by HUGE is the known interaction of *FLT3*-mutant genotypes sensitive to *FLT3* inhibitors, such as Midostaurin. This fact is only an example of the key importance of considering the HUGE effect when analyzing LEDs with large-scale functional screens. 

A *p*-value histogram can be modeled as the superposition of two distributions, a uniform distribution (which corresponds to the null hypothesis) and another distribution with a larger proportion of low *p*-values. A good covariate splits the overall *p*-value histogram into histograms with different enrichments in small *p*-values. If all the histograms related to a covariate have similar shapes, it means that the covariate is uninformative. Here, we show that stating which gene is mutated in each test is a good covariate for the LED prediction problem because there is a hub effect of gene aberrations in gene essentiality. The usage of covariates has successfully been incorporated before in other genomics applications (e.g., the abundance of a gene is known to be informative in differential expression analyses; or the proximity of loci in the genome is known to play a role in genome-wide association studies), but it has not yet been exploited in large-scale functional genomic screens.

One main limitation lies in the volume of data required for its execution due to the need for multiple hypotheses to detect the Hub-Effect. Hence, the HUGE-based approach will not obtain such striking results if applied to the analysis of smaller experiments in number, it would perform similarly to current standard methods. Nevertheless, this method was developed for large-scale screening analyses.

We are confident that the HUGE-based approach to calculating LEDs has great potential if applied to the study of patient data. Nowadays, drug development usually starts from large-scale loss-of-function screenings. Therefore, this work has identified a large number of LEDs across 19 tumor types in three different large-scale experiments. Moreover, to facilitate the in vitro validation of these LEDs as possible therapeutic targets, we added information regarding targeted drugs for those essential genes that are drug targets.

Predicting true LEDs is especially challenging for tumors with high genetic heterogeneity. In AML, for instance, state-of-the-art approaches only recover two LEDs. The HUGE-based approach captured 24 LEDs for the same False Discovery Rate (FDR). Interestingly, *NRASwt-PTPN11* LED, which was only identified by HUGE, was validated in vitro. The validation in AML highlights the potential of the HUGE-based approach to discover and validate new LEDs of biomarkers and drug targets. We pinpoint the *dLED* characteristic of the *NRAS* gene, meaning that if a tumor has *NRAS* mutated a treatment that targets *NRAS* itself would be the best option to reduce their tumorigenicity, whereas if it is *NRAS* wild-type, a *PTPN11* inhibition would be a better recommendation. This *dLED* discovery confers special relevance to clinically translational therapeutic strategies, as it was proved effective in AML cell lines, further validation in ex vivo analysis and murine models is required but if the result is effective, it could be suggested as a treatment and it could incentivize drug development targeting *NRAS* and *PTPN11*. This methodology has potential applications both in basic and clinical research.

## 5. Conclusions

In conclusion, this work provides a computational approach to identifying LEDs with increased predictive power. This analysis opens new possibilities for the use of genetic variants as predictive events for precision oncology, by analyzing both previous and future functional genomic screens. Moreover, this analysis enhances current applications in translational oncology, such as drug development or drug repositioning projects.

## Figures and Tables

**Table 1:** **Ranking of lethal dependencies in AML using the covariate-based statistical approach.** The ranking is divided into three groups regarding the typology of the lethal dependency relationship: Positive Lethal Dependency (PLD), Negative Lethal Dependency (NLD) or Dual Lethal Dependency (DLD) (Figure S1). The Increment of Essentiality column represents the average variation in the DEMETER score between altered and wild-type cells, and its sign is related to the lethal dependency relationship. Lethal dependencies that share the same essential gene and the same Increment of Essentiality sign were omitted in this table (see complete data in Supplementary Table S3).

Gene Variant Biomarker	Essential Gene	Increment of Essentiality	t-Score	*p*-Value	Local FDR
Positive Lethal Dependencies					
TGS1	SNRPF	−7.87	−4.05	6.69 × 10^−4^	3.36 × 10^−1^
CLTCL1	UBR5	−6.66	−3.59	1.99 × 10^−3^	2.20 × 10^−1^
FLT3	FLT3	−6.36	−4.53	2.28 × 10^−4^	2.00 × 10^−1^
CDK14	CDK2	−3.95	−2.75	1.28 × 10^−2^	4.30 × 10^−1^
AURKC	ACTL6A	−3.26	−3.89	9.55 × 10^−4^	4.99 × 10^−1^
Negative Lethal Dependencies					
NPM1	EEF2	3.81	3.34	3.39 × 10^−3^	5.96 × 10^−1^
PIK3C2G	CDK6	3.35	2.95	8.20 × 10^−3^	3.51 × 10^−1^
NCOA3	EP300	3.04	2.75	1.25 × 10^−2^	4.94 × 10^−1^
CDK14	CCND2	2.97	2.22	3.88 × 10^−2^	4.99 × 10^−1^
EPHB6	ZNF266	2.53	2.77	1.22 × 10^−2^	3.42 × 10^−1^
ZFYVE9	TOM1L2	2.14	2.35	2.96 × 10^−2^	5.12 × 10^−1^
Dual Lethal Dependencies					
NRAS	NRAS	−6.83	−8.71	4.67 × 10^−8^	1.38 × 10^−4^
NRAS	PTPN11	4.17	2.2	4.05 × 10^−2^	5.89 × 10^−1^
EP300	PLK1	−8.11	−4.04	7.01 × 10^−4^	2.17 × 10^−1^
EP300	KLF2	3.69	4.08	6.38 × 10^−4^	2.12 × 10^−1^

## Data Availability

All the graphs showing lethal dependencies for the 19 tumor types analyzed can be visualized in the following interactive tool https://fcarazo.shinyapps.io/visnetShiny/ (accessed on 24 June 2022). All genes and biomarkers predicted in this study can be downloaded from Tables S7–S24.

## Supplementary Materials

The following are available online at https://www.mdpi.com/article/10.3390/cancers14133251/s1, Supplementary Methods: Section S1: Cell Culture, Section S2: Cell Transfection, Section S3: Cell proliferation assay, Section S4: Quantitative-PCR(Q-PCR), Section S5: Statistical pipeline, Section S6: A larger number of positives outperforms specificity and sensitivity. Supplementary Figures: Figure S1: Types of Lethal Dependencies, Figure S2: Computational pipeline to find lethal dependencies, Figure S3: Schematic representation of the covariate-based statistical approach in this context, Figure S4: Histogram of *p*-values of all LEDs in AML, Figure S5: Histogram of *p*-values of all lethal dependencies in acute myeloid leukemia vs. *p*-values associated with each gene variant, Figure S6: Histogram of *p*-values oflethal dependencies in breast cancer vs. *p*-values associated with each gene variant, Figure S7: Histogram of *p*-values of all lethal dependencies in central nervous system astrocytoma grade IV vs. *p*-values associated with each gene variant, Figure S8: Histogram of *p*-values of all lethal dependencies in colon adenocarcinoma vs. *p*-values associated with each gene variant, Figure S9: Histogram of *p*-values of all lethal dependencies in upper aerodigestive tract squamous cell carcinoma vs. *p*-values associated with each gene variant, Figure S10: Histogram of *p*-values of all lethal dependencies in diffuse large B-cell lymphoma vs. *p*-values associated with each gene variant, Figure S11: Histogram of *p*-values of all lethal dependencies in esophagus squamous cell carcinoma vs. *p*-values associated with each gene variant, Figure S12: Histogram of *p*-values of all lethal dependencies in lung large cell carcinoma vs. *p*-values associated with each gene variant, Figure S13: Histogram of *p*-values of all lethal dependencies in lung adenocarcinoma vs. *p*-values associated with each gene variant, Figure S14: Histogram of *p*-values of all lethal dependencies in lung squamous cell carcinoma vs. *p*-values associated with each gene variant, Figure S15: Histogram of *p*-values of all lethal dependencies in multiple myeloma vs. *p*-values associated with each gene variant, Figure S16: Histogram of *p*-values of all lethal dependencies in non–small cell lung carcinoma vs. *p*-values associated with each gene variant, Figure S17: Histogram of *p*-values of all lethal dependencies in osteosarcoma vs. *p*-values associated with each gene variant, Figure S18: Histogram of *p*-values of all lethal dependencies in ovary adenocarcinoma vs. *p*-values associated with each gene variant, Figure S19: Histogram of *p*-values of all lethal dependencies in pancreas ductal carcinoma vs. *p*-values associated with each gene variant, Figure S20: Histogram of *p*-values of all lethal dependencies in small cell lung carcinoma vs. *p*-values associated with each gene variant, Figure S21: Histogram of *p*-values of all lethal dependencies in skin carcinoma vs. *p*-values associated with each gene variant, Figure S22: Histogram of *p*-values of all lethal dependencies in stomach adenocarcinoma vs. p-values associated with each gene variant, Figure S23: Histogram of *p*-values of all lethal dependencies in uterine corpus endometrial carcinoma vs. *p*-values associated with each gene variant, Figure S24: The number of LEDs found (FDR ≤ 20%), Figure S25: ROC and precision-recall curves of four tumor types, Figure S26: Volcano plot of Synthetic lethal genes related to NRAS-mutated (A) and EP300-mutated (B) phenotypes, Figure S27: mRNA expression of NRAS and PTPN11 genes after nucleofection with the specific siRNAs. Supplementary Tables: Table S1: Associations within the top 500 pairs predicted using the HUGE-based and standard pipeline algorithms in AML that match the knowledgebase of clinical interpretations of somatic genomic variants in cancer of the Variant Interpretation for Cancer Consortium (VICC), Table S2: Essential genes for AML. Selected genes meet the following criteria: (i) must be essential in ≥25% of AML cell lines (DEMETER essentiality threshold set to -2), Table S3: Complete ranking of lethal dependencies in AML using the HUGE-based statistical approach. The Increment of Essentiality (deltaEs) column represents the average variation in the DEMETER score between altered and wild-type cells, and its sign is related to the lethal dependy relationship, Table S4: Cell lines included in the analysis, Table S5: AML cell lines included in the analysis, Table S6: Ranking of pairs mutation biomarker and essential genes in 19 tumor types using a covariate-based statistical model, Table S7: Ranking of pairs mutation biomarker and essential genes in OS, Table S8: Ranking of pairs mutation biomarker and essential genes in BRCA, Table S9: Ranking of pairs mutation biomarker and essential genes in CNSA-IV, Table S10: Ranking of pairs mutation biomarker and essential genes in UCEC, Table S11: Ranking of pairs mutation biomarker and essential genes in COAD, Table S12: Ranking of pairs mutation biomarker and essential genes in DLBCL, Table S13: Ranking of pairs mutation biomarker and essential genes in MM, Table S14: Ranking of pairs mutation biomarker and essential genes in LUAD, Table S15: Ranking of pairs mutation biomarker and essential genes in LCC, Table S16: Ranking of pairs mutation biomarker and essential genes in NSCLC, Table S17: Ranking of pairs mutation biomarker and essential genes in SCLC, Table S18: Ranking of pairs mutation biomarker and essential genes in LUSC, Table S19: Ranking of pairs mutation biomarker and essential genes in ESCA, Table S20: Ranking of pairs mutation biomarker and essential genes in OVAD, Table S21: Ranking of pairs mutation biomarker and essential genes in PDAC, Table S22: Ranking of pairs mutation biomarker and essential genes in SKCM, Table S23: Ranking of pairs mutation biomarker and essential genes in STAD, Table S24: Ranking of pairs mutation biomarker and essential genes in STAD.
