# Supplementary material for: Identifying Lethal Dependencies with HUGE Predictive Power
Source: Cancers (Basel). 2022 Jul 1;14(13):3251. doi: 10.3390/cancers14133251 (PMC9264916; doi:10.3390/cancers14133251)
Supplement: Supplementary file 1 [file cancers-14-03251-s001.zip › 2022_Gimeno, San Jose, et al. Suppl. Figures.pdf]

Supplementary figures

Identifying Lethal Dependencies with HUGE Predictive Power

Marian Gimeno[1,‡], Edurne San José-Enériz[2,3,‡], Angel Rubio[1,4], Leire Garate[3,5], Estíbaliz Miranda[2,3], Carlos Castilla[1], Xabier Agirre[2,3,*], Felipe Prosper[2,3,5,*], and Fernando Carazo[1,4*].

[1]Departamento de Ingeniería Biomédica y Ciencias, TECNUN, Universidad de Navarra, San Sebastian, Spain.

[2]Programa Hemato-Oncología, Centro de Investigación Médica Aplicada, IDISNA, Universidad de Navarra, Pamplona, Spain.

[3]Centro de Investigación Biomédica en Red de Cáncer (CIBERONC), Madrid, Spain.

[4]Instituto de Ciencia de los Datos e Inteligencia Artificial (DATAI), Universidad de Navarra, 31080 Pamplona, Spain

[5]Departamento de Hematología, Clinica Universidad de Navarra, Universidad de Navarra, Pamplona.

‡MG and ESJ-E contributed equally to this work.

*These authors share senior authorship. Correspondence and requests for materials should be addressed to F.P. (email: fprosper@unav.es), F.C. (email: fcarazo@tecnun.es) and X.A. (email: xaguirre@unav.es).

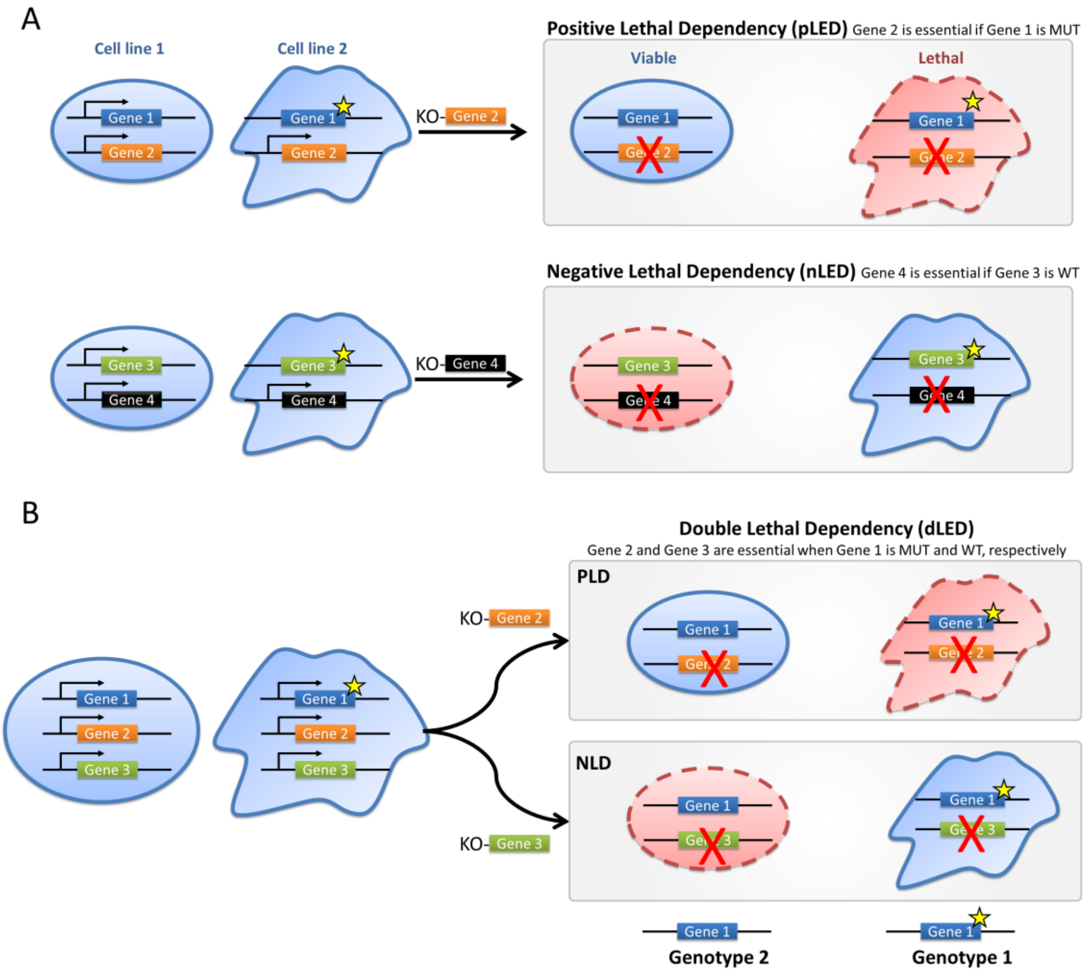

Figure S1. Types of Lethal Dependencies. Lethal dependencies that affect two genes: (A) in a Positive Lethal Dependency (pLED), a gene is essential for tumor survival when another gene is mutated (MUT). This is the traditional concept of synthetic lethality, in which a gene knockout (KO) causes cellular death only for another gene's mutant phenotype. (B) Conversely, in Negative Lethal Dependency (nLED), a gene is essential for tumor survival when another gene is not genetically altered (wild type-WT), here gene variant confers resistance to the inhibition. (C) A lethal dependency that affects three genes: Dual Lethal Dependency (dLED), an altered gene (Gene 1) confers, at the same time, sensitivity to the inhibition of one gene (Gene 2) and resistance to the inhibition of another gene (Gene 3). In this figure, the shape of the cells denotes different cell-types with different genomic characteristics. The color of the cells denote whether the cell survives to the knock down or not. The star shape in a gene denotes a genetic variant. The red crosses denote pharmacological inhibition.

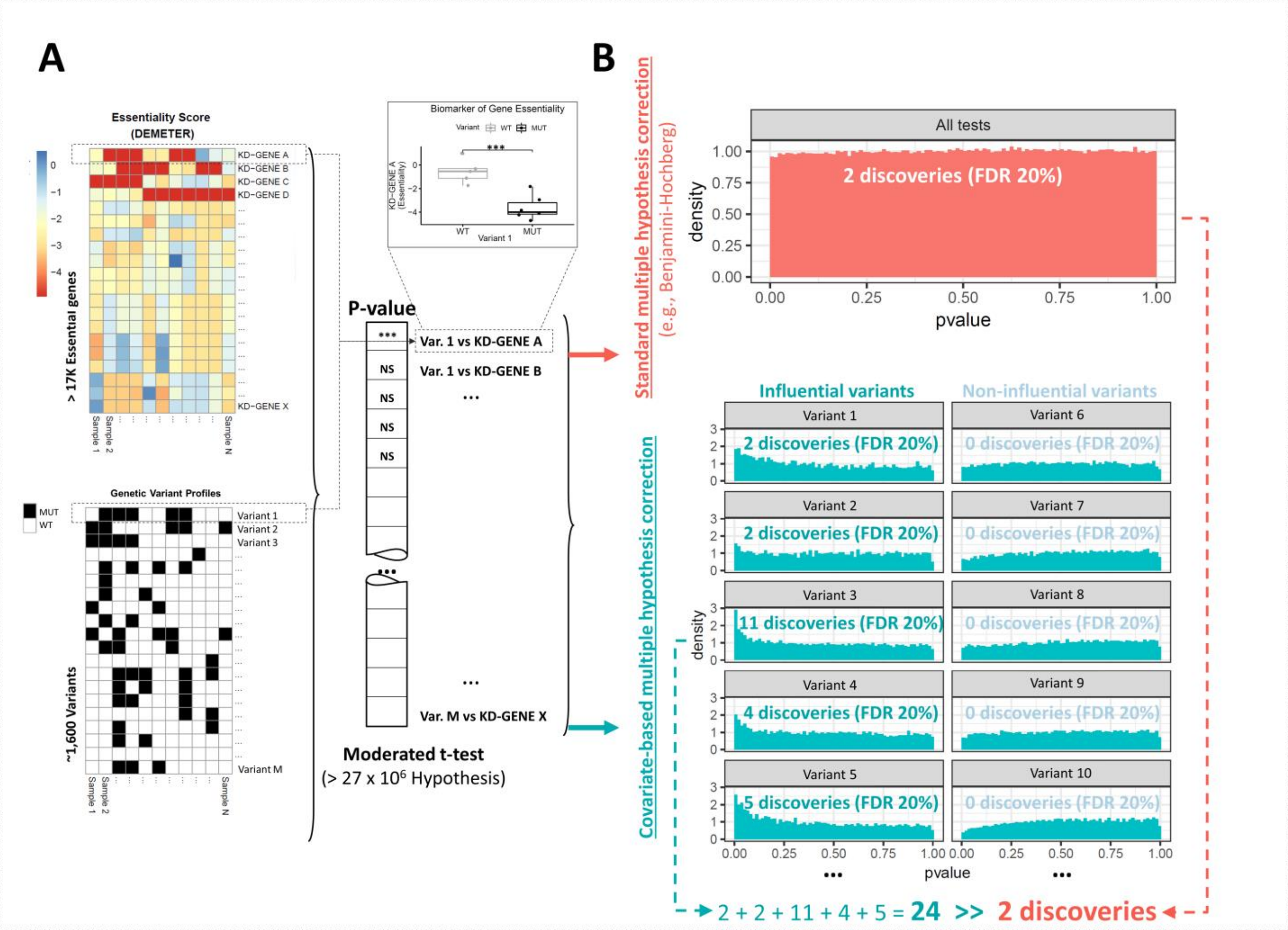

Figure S2. Computational pipeline to find lethal dependencies. (A) Scheme of data integration for N samples (cell lines). RNAi libraries data (gene essentiality; DEMETER score) and gene variant panels are represented as two heatmaps. Each pair of a knock-down gene (KD-gene) and a gene variant defines a p-value, which represents a lethal dependency. Boxplots represent the DEMETER score of a cell line when inhibiting one gene (X-axis) depending on the genetic alteration of another gene (Y-axis). Genes with a DEMETER score < -2 are considered essential for a cell line. (B) Scheme of the histogram of P-values using standard approaches (e.g., Storey-Tibshirani), in red; and using a covariate-based algorithm, in blue.

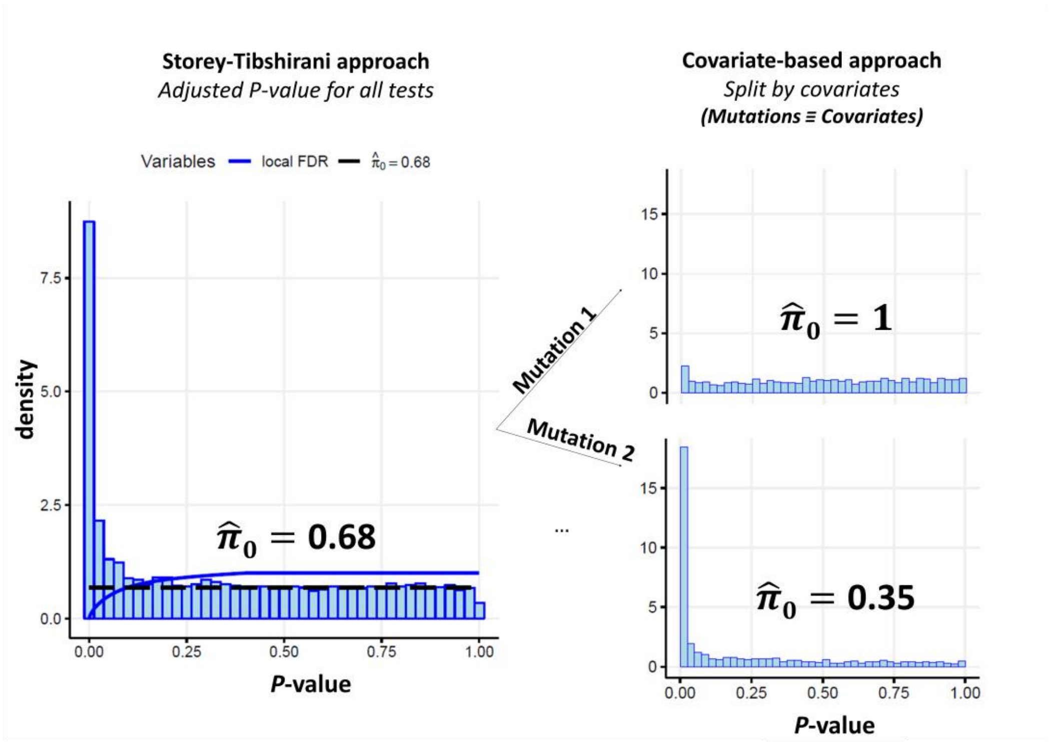

Figure S3. Schematic representation of the covariate-based statistical approach in this context. In this case, the use of genetic variants as covariates of a covariate-based problem allows the reduction of false positive rate, and consequently, the percentage of true null-hypothesis in a statistical test.

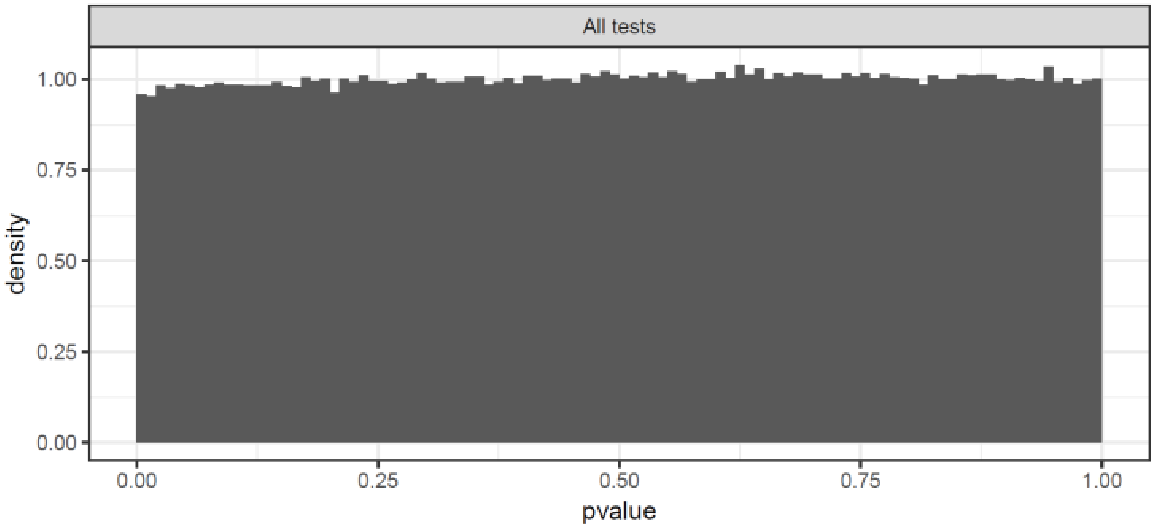

Figure S4. Histogram of P-values of all lethal dependencies in acute myeloid leukemia. Previous efforts to correct multiple testing in this problem consider a single set of tests (all gene aberrations and CRISPR-Cas9 knockouts) and apply a correction that control the FDR, such as Storey-Tibshirani (ST), as done in the Project Score. Interestingly, in this approach histogram of p-values shows flat-shaped histograms.

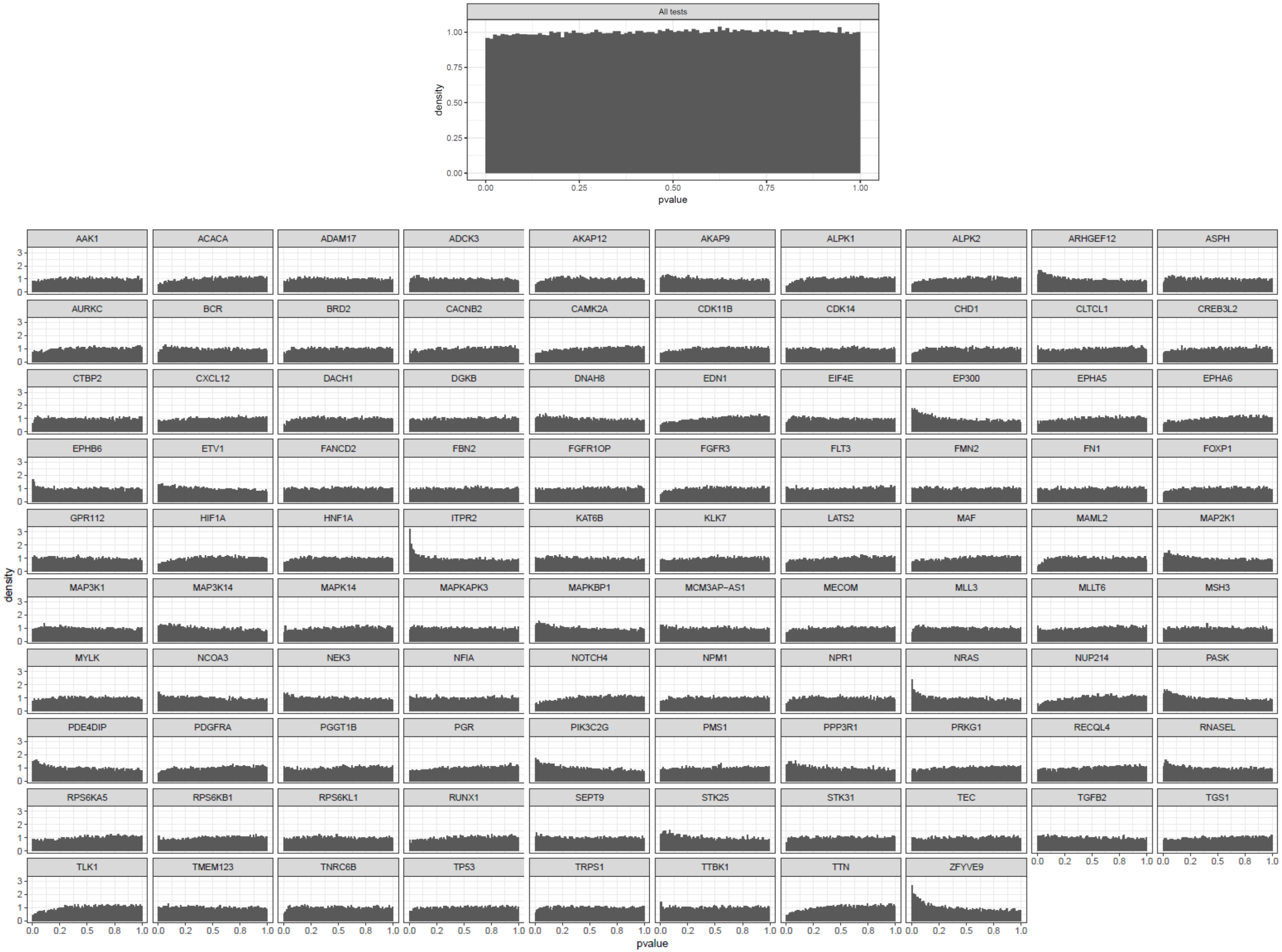

Figure S5. Histogram of P-values of all lethal dependencies in acute myeloid leukemia vs p-values associated with each gene variant.

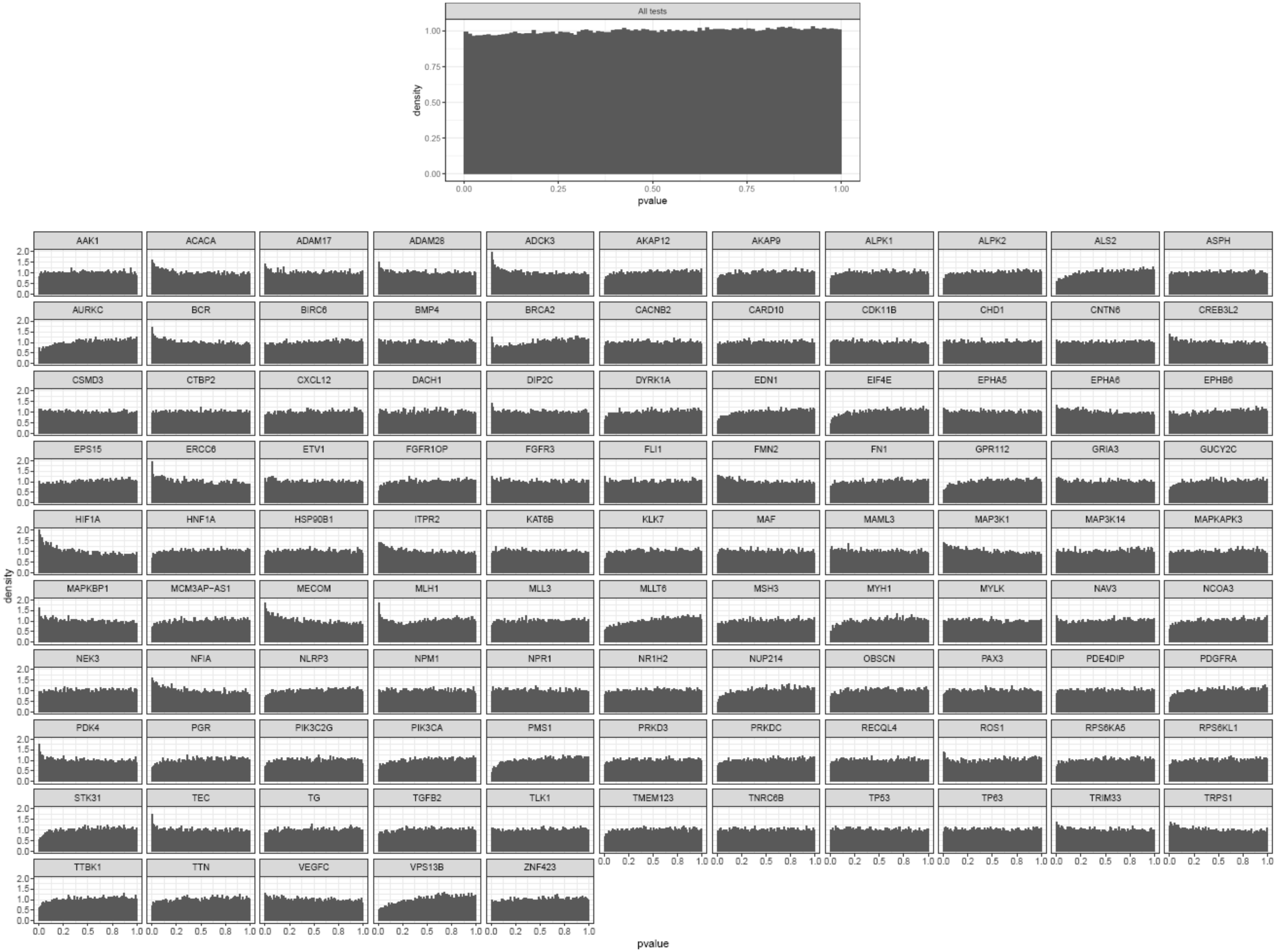

Figure S6. Histogram of P-values oflethal dependencies in breast cancer vs p-values associated with each gene variant.

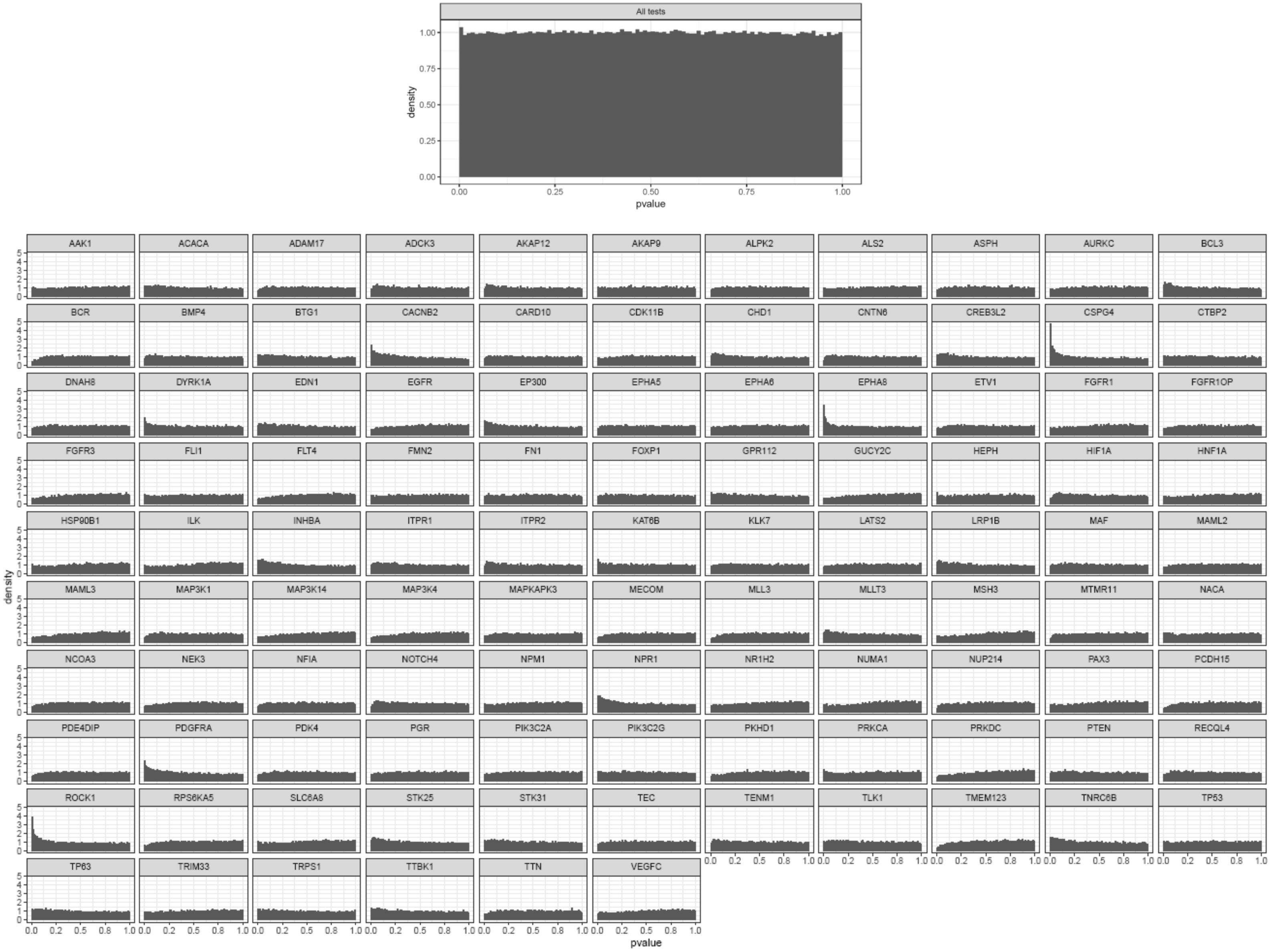

Figure S7. Histogram of P-values of all lethal dependencies in central nervous system astrocytoma grade IV vs p-values associated with each gene variant.

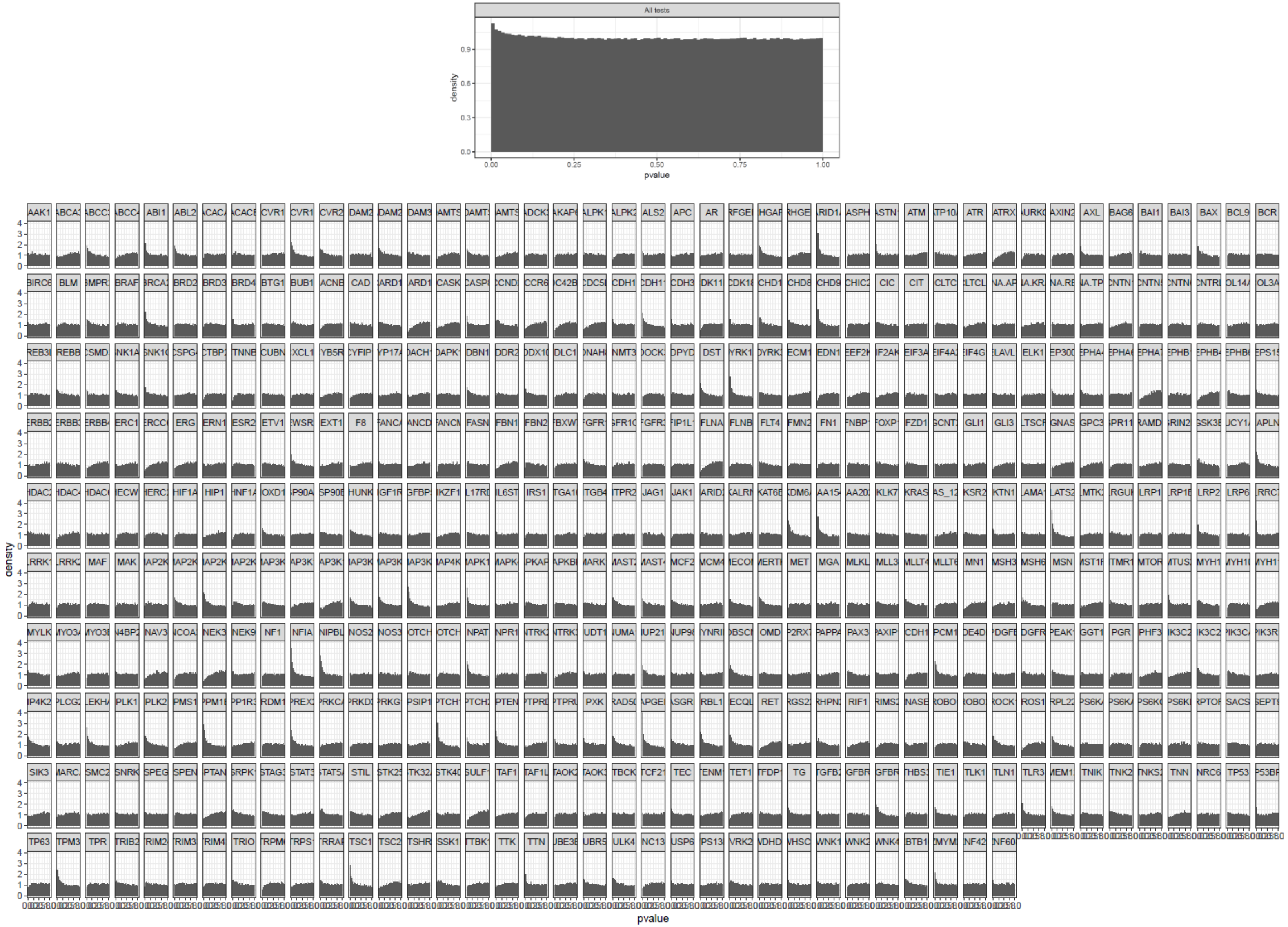

Figure S8. Histogram of P-values of all lethal dependencies in colon adenocarcinoma vs p-values associated with each gene variant.

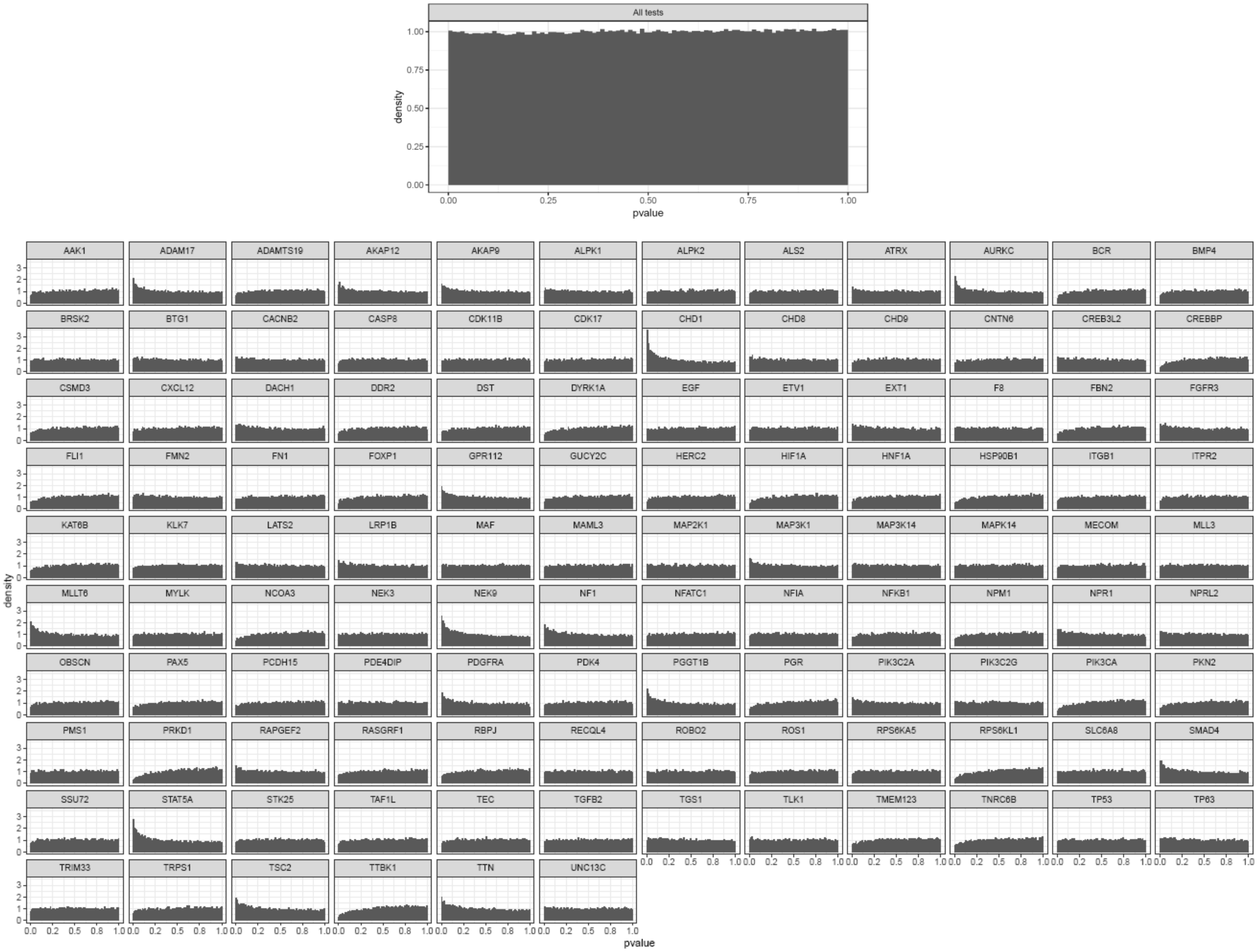

Figure S9. Histogram of P-values of all lethal dependencies in upper aerodigestive tract squamous cell carcinoma vs p-values associated with each gene variant.

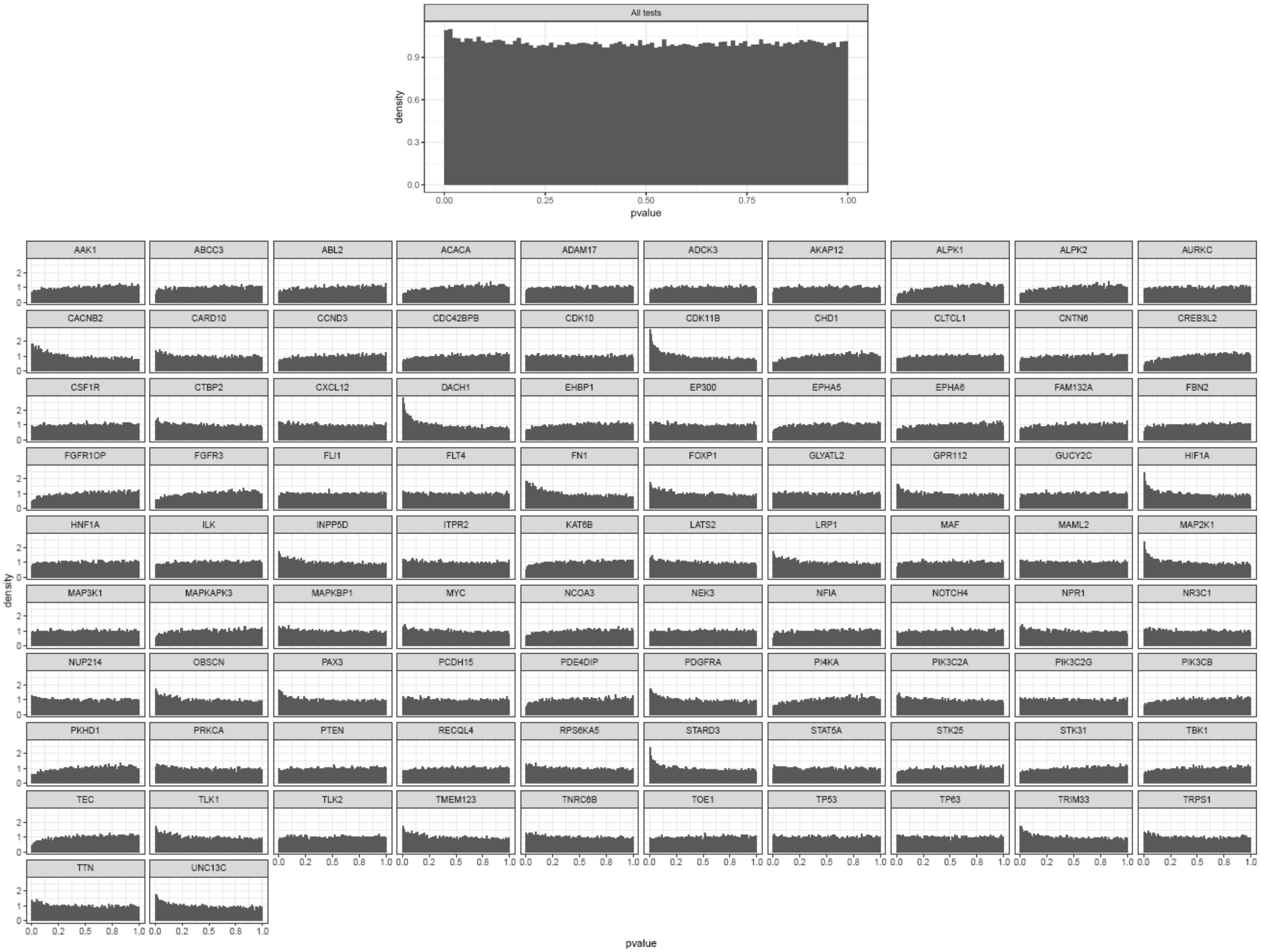

Figure S10. Histogram of P-values of all lethal dependencies in diffuse large B-cell lymphoma vs p-values associated with each gene variant.

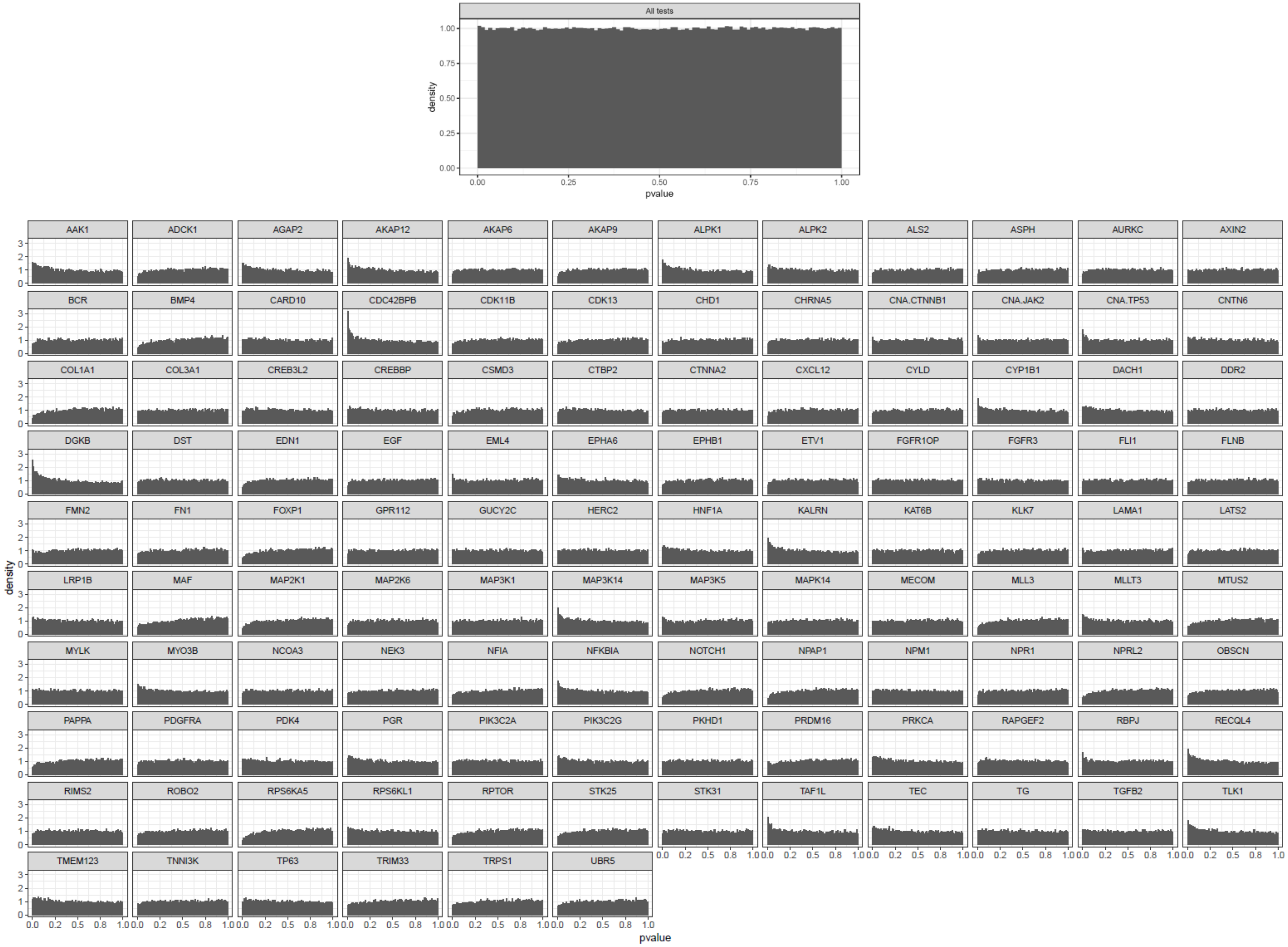

Figure S11. Histogram of P-values of all lethal dependencies in esophagus squamous cell carcinoma vs p-values associated with each gene variant.

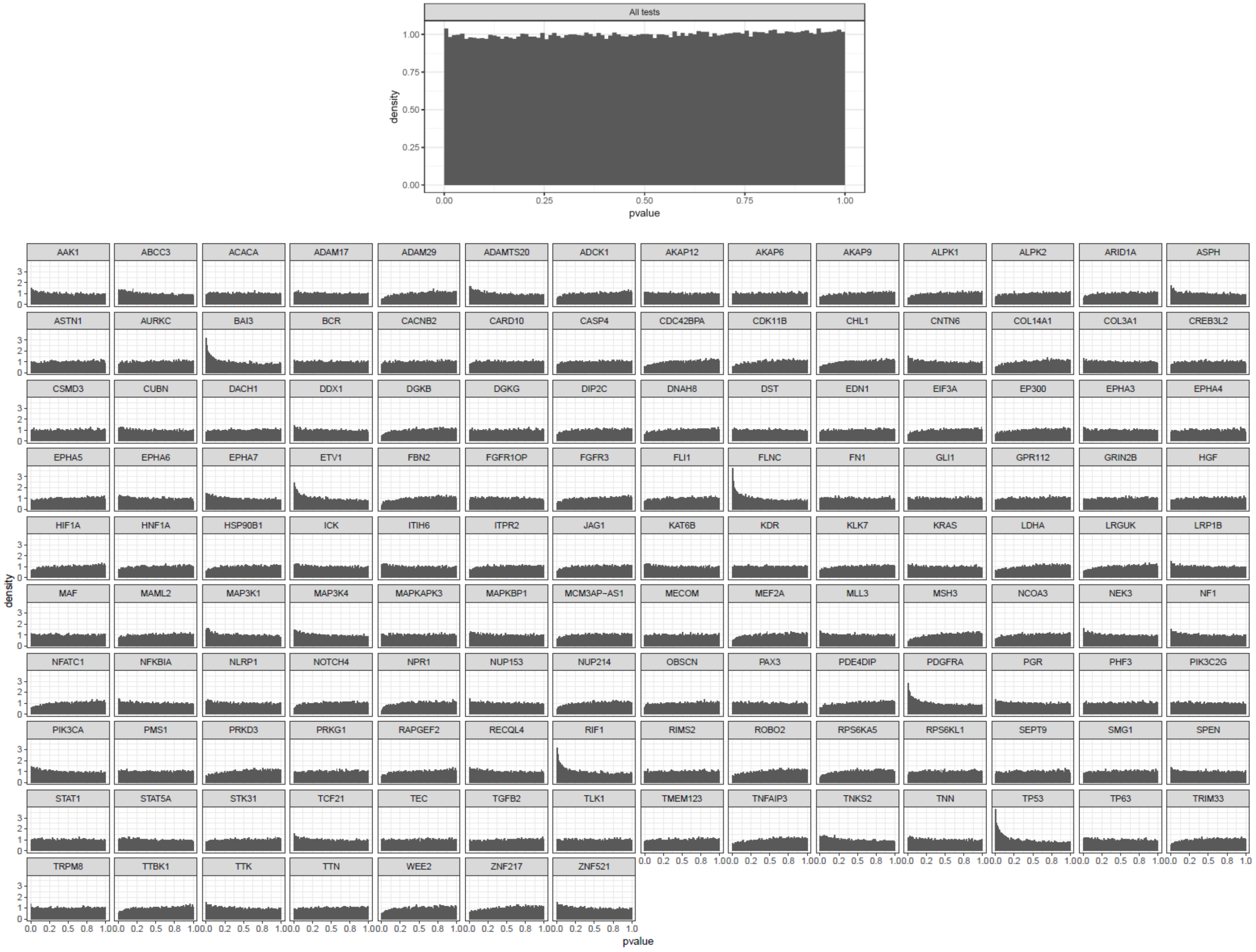

Figure S12. Histogram of P-values of all lethal dependencies in lung large cell carcinoma vs p-values associated with each gene variant.

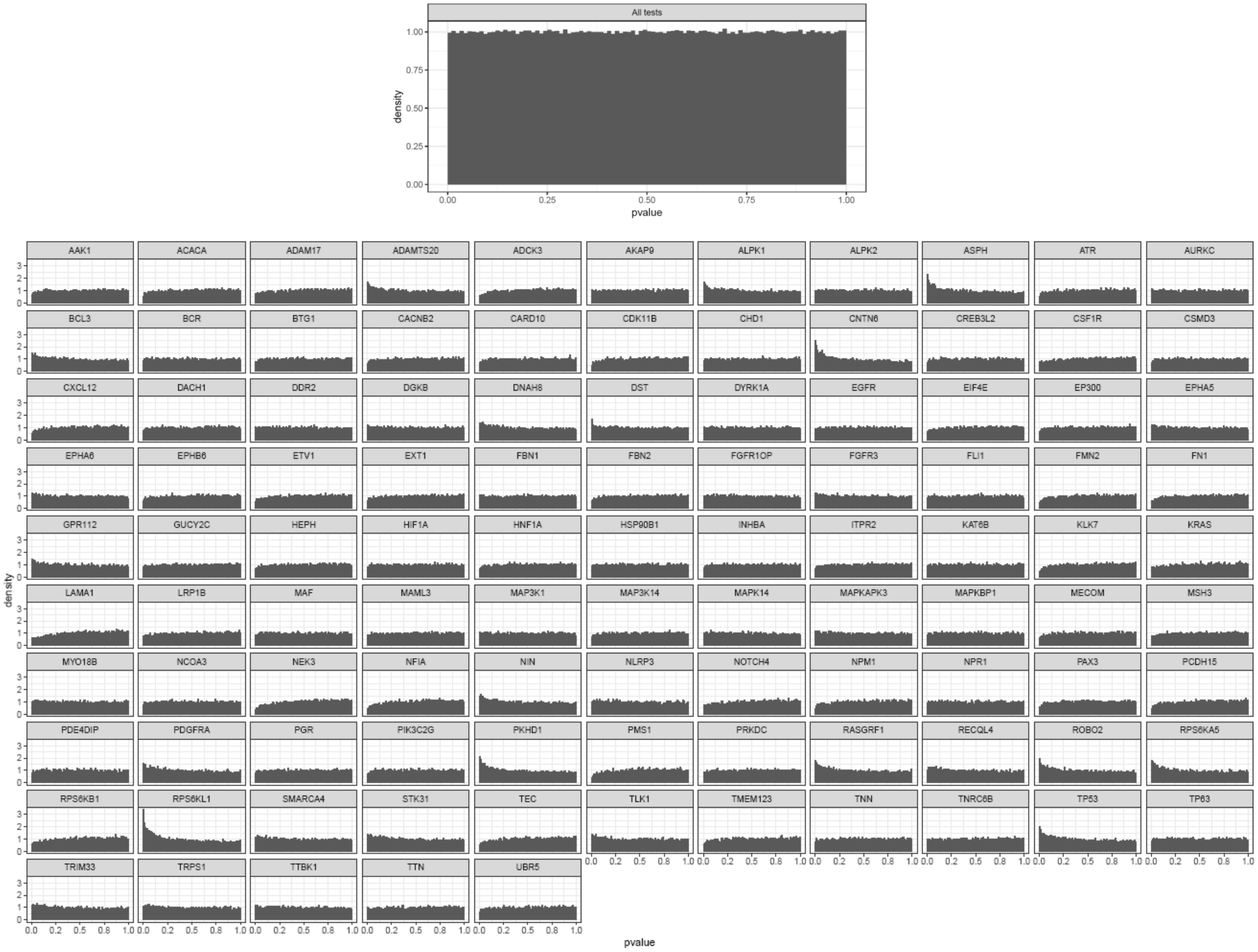

Figure S13. Histogram of P-values of all lethal dependencies in lung adenocarcinoma vs p-values associated with each gene variant.

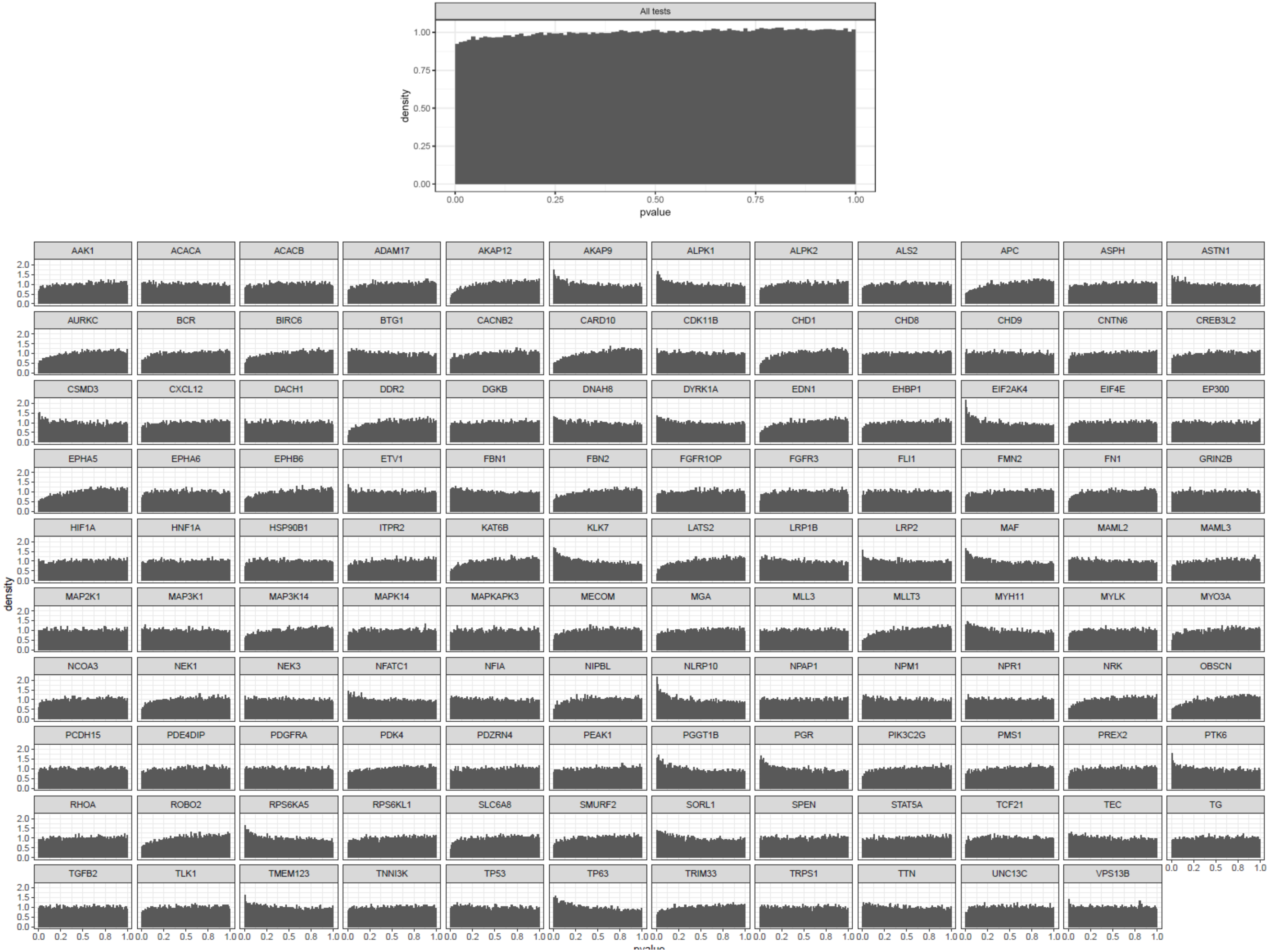

Figure S14. Histogram of P-values of all lethal dependencies in lung squamous cell carcinoma vs p-values associated with each gene variant.

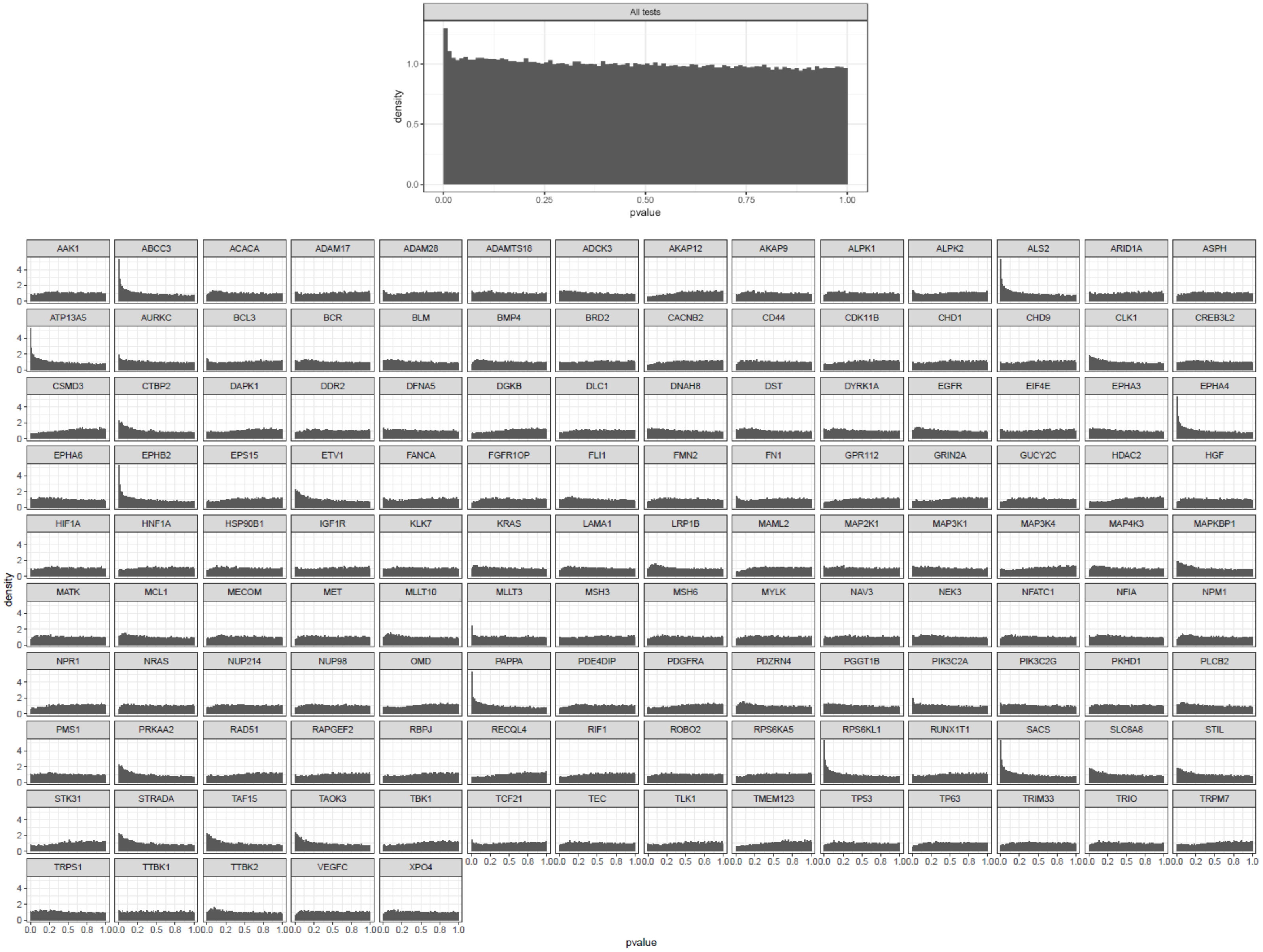

Figure S15. Histogram of P-values of all lethal dependencies in multiple myeloma vs p-values associated with each gene variant.

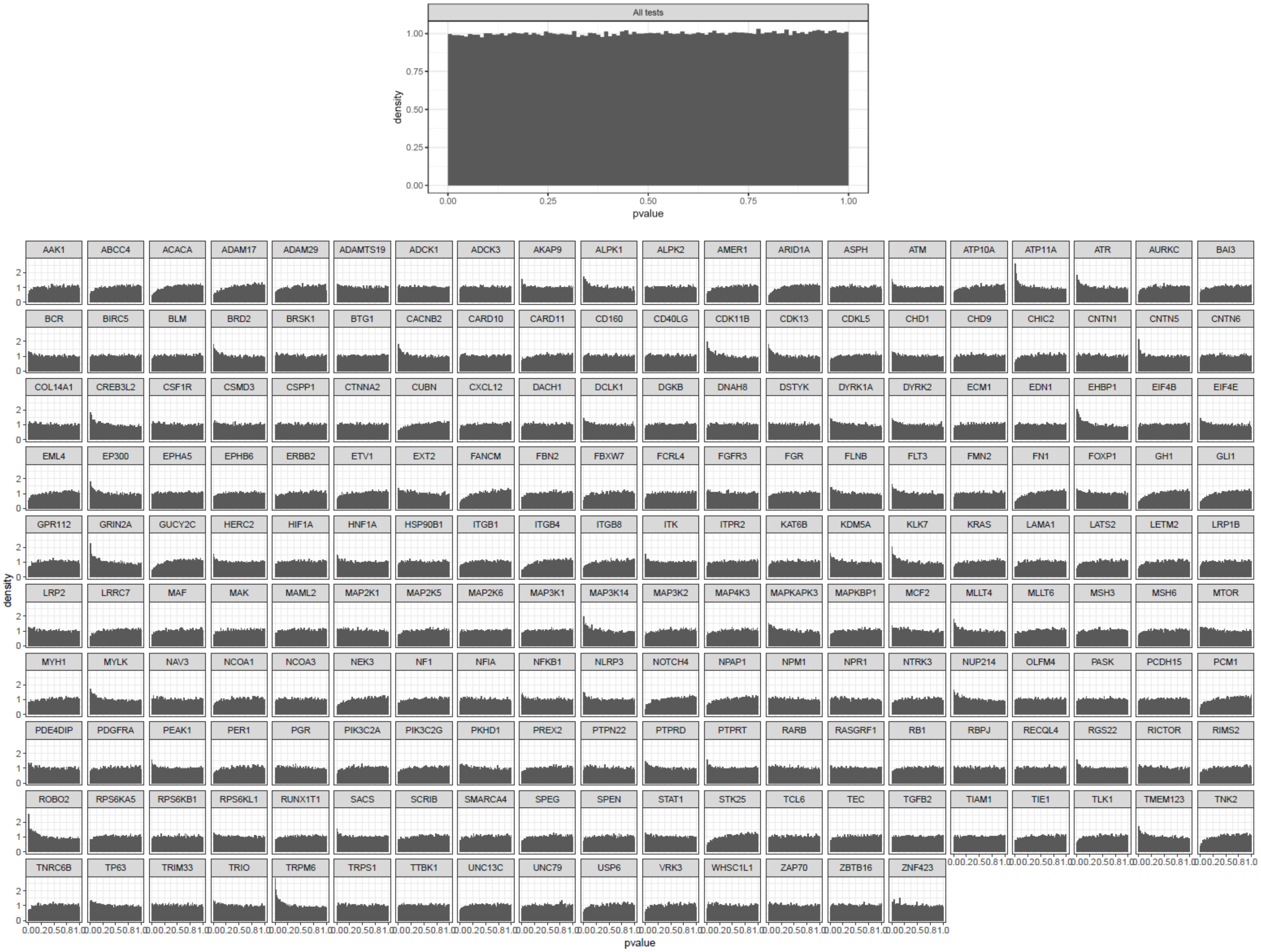

Figure S16. Histogram of P-values of all lethal dependencies in non–small cell lung carcinoma vs p-values associated with each gene variant.

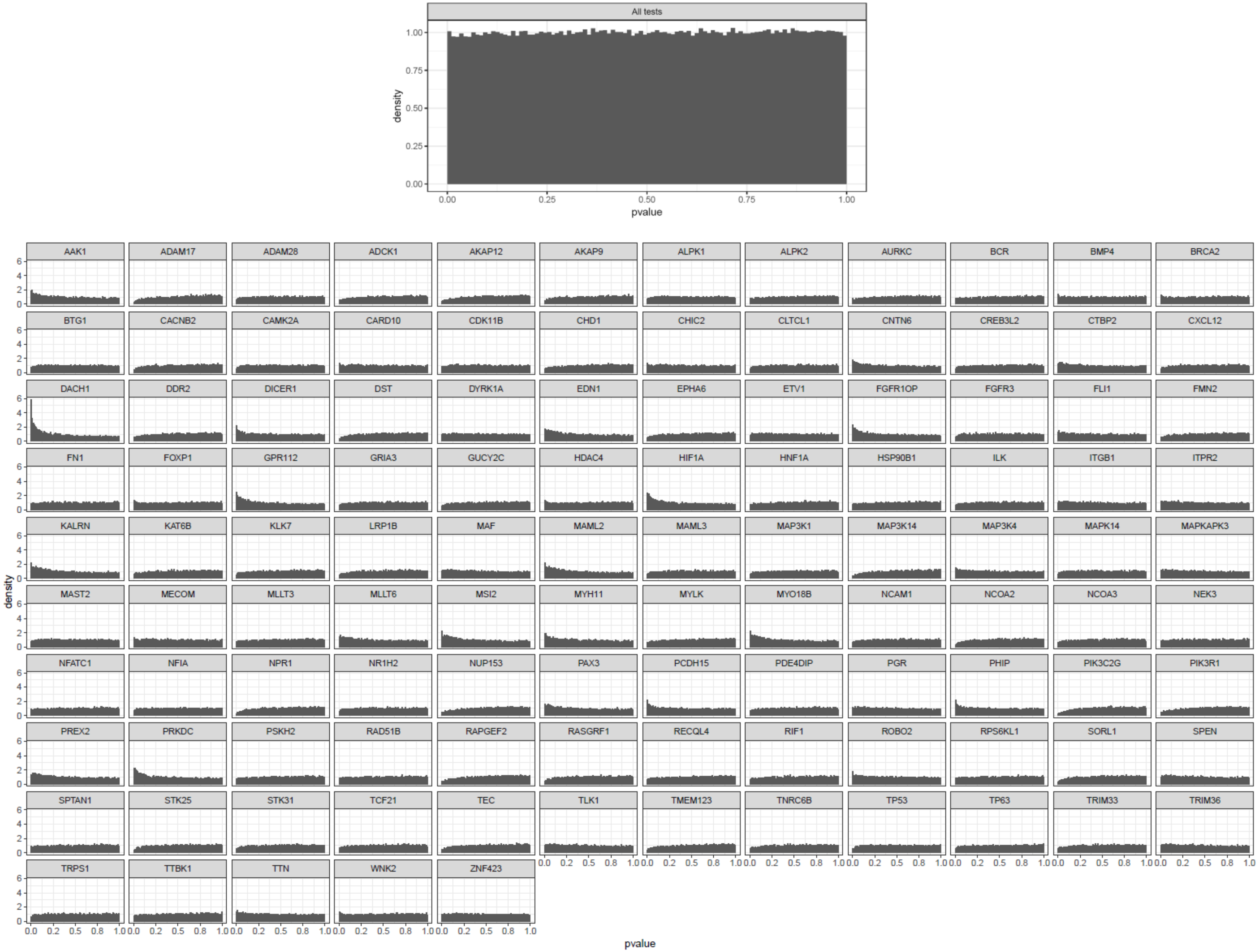

Figure S17. Histogram of P-values of all lethal dependencies in osteosarcoma vs p-values associated with each gene variant.

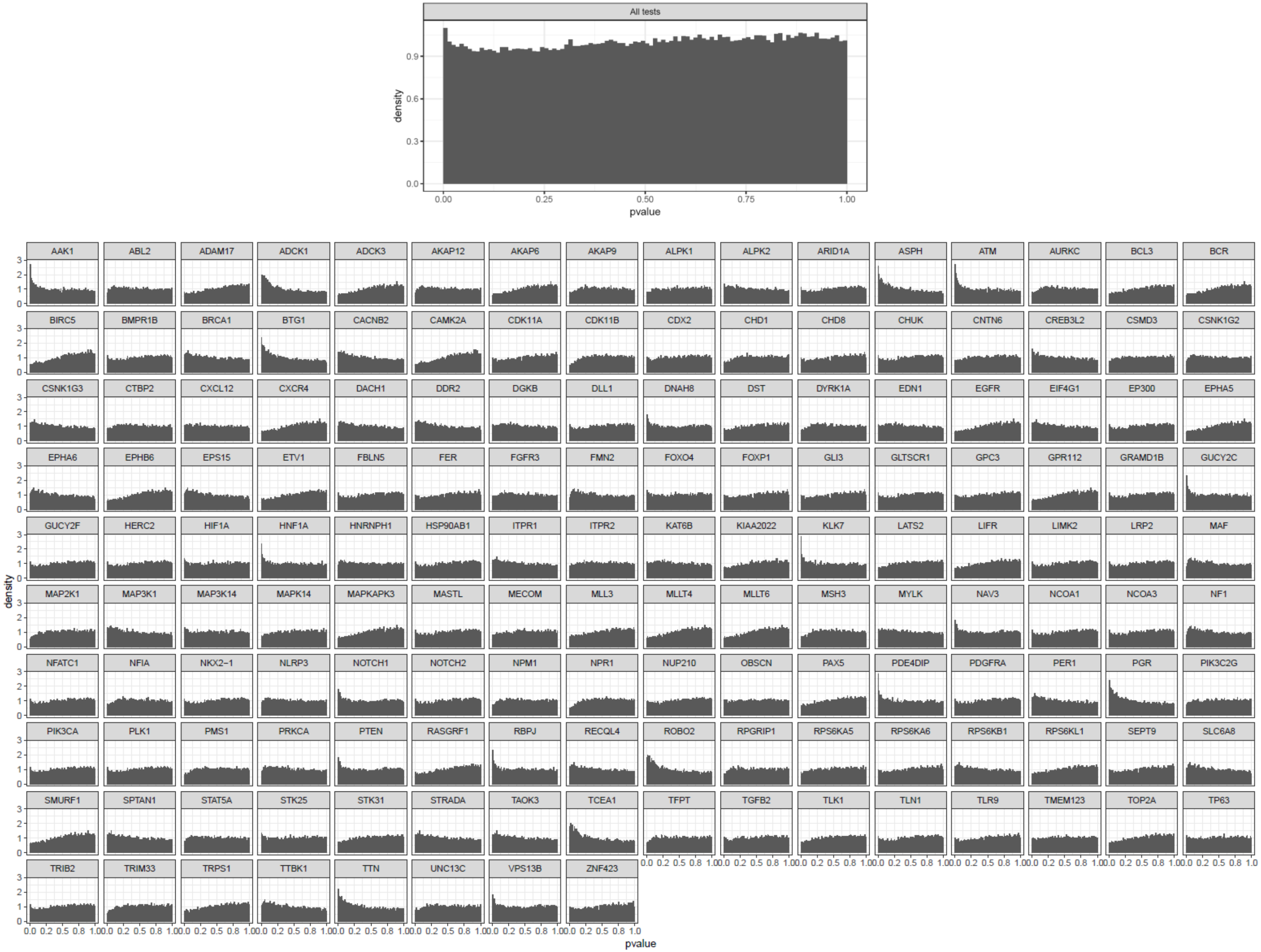

Figure S18. Histogram of P-values of all lethal dependencies in ovary adenocarcinoma vs p-values associated with each gene variant.

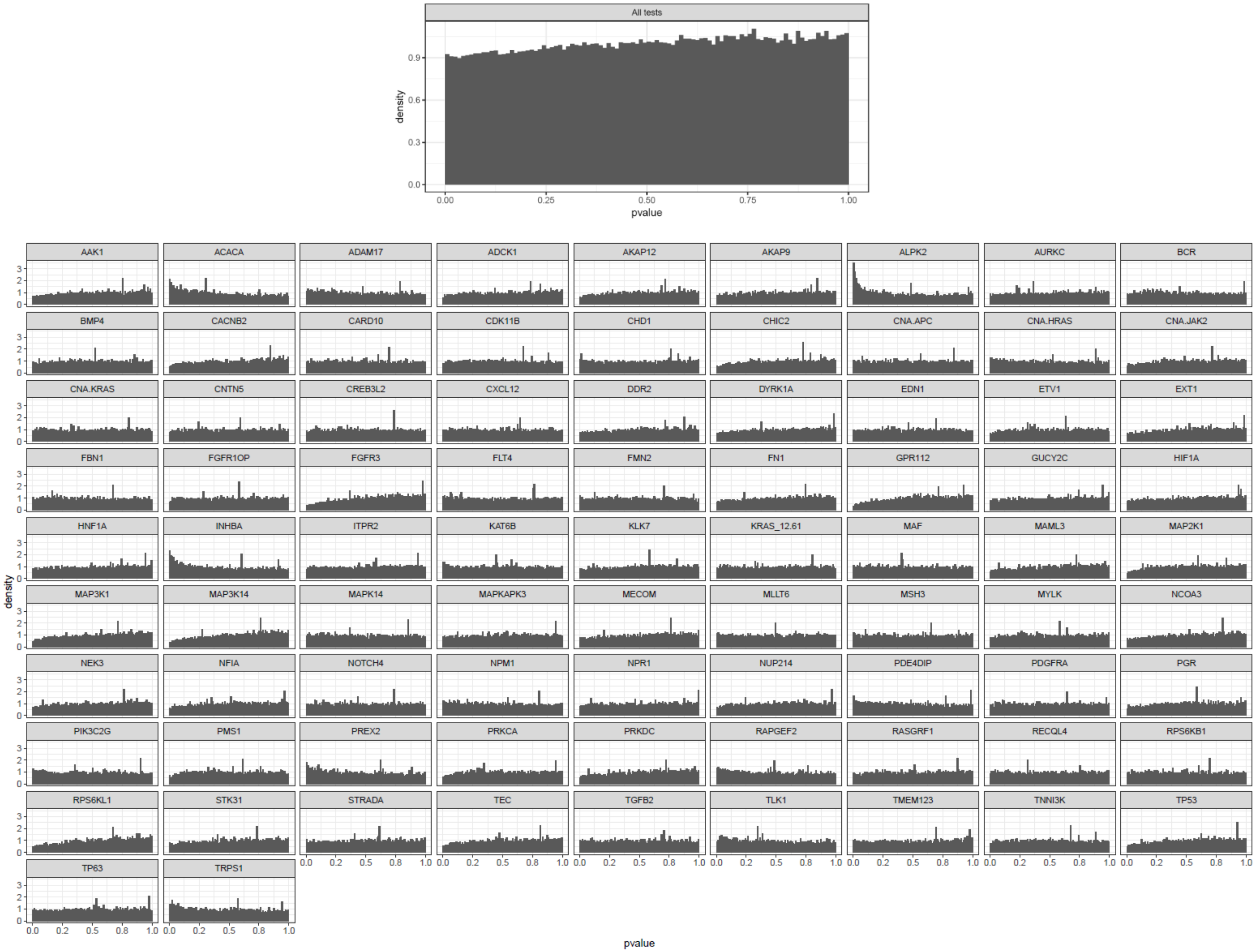

Figure S19. Histogram of P-values of all lethal dependencies in pancreas ductal carcinoma vs p-values associated with each gene variant.

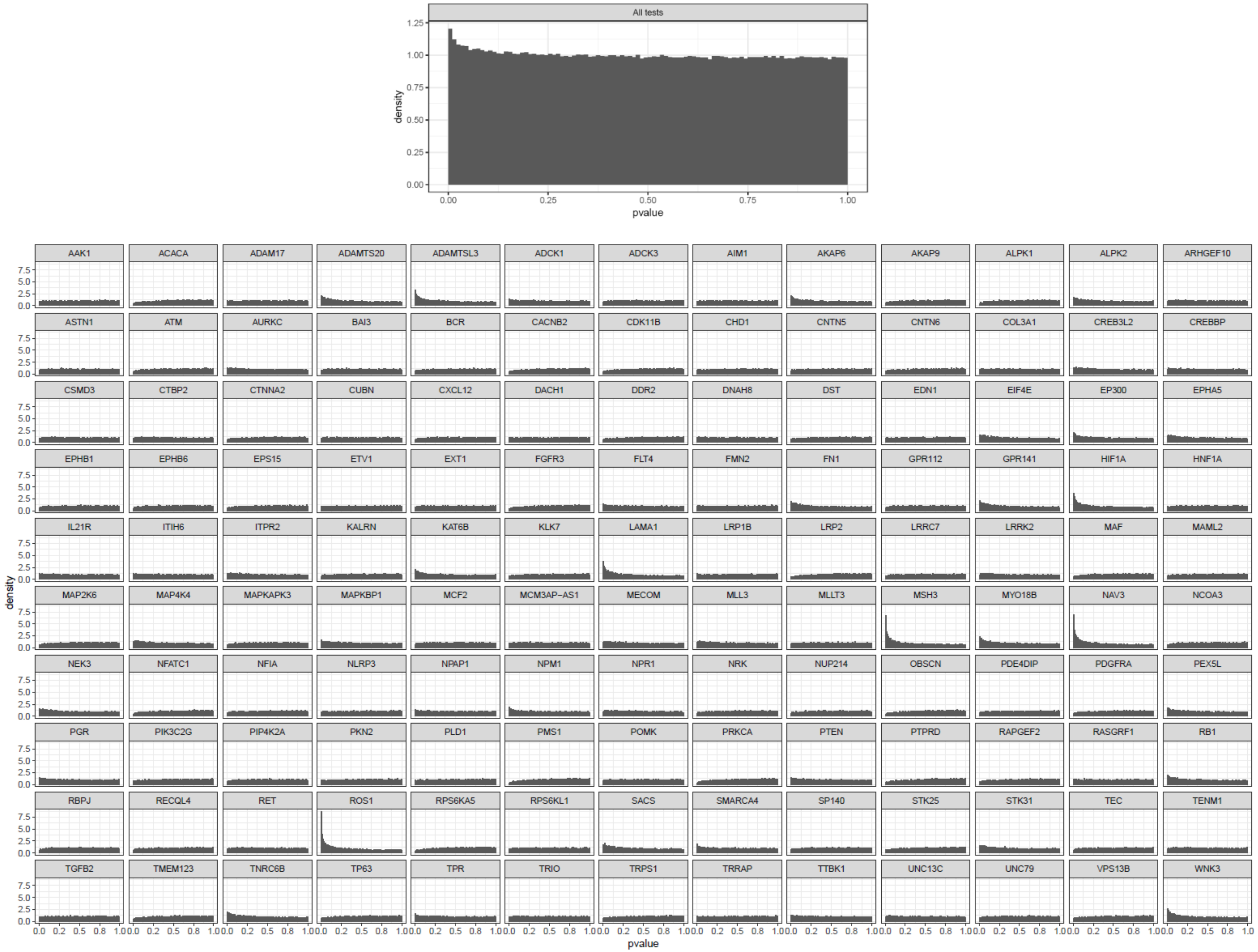

Figure S20. Histogram of P-values of all lethal dependencies in small cell lung carcinoma vs p-values associated with each gene variant.

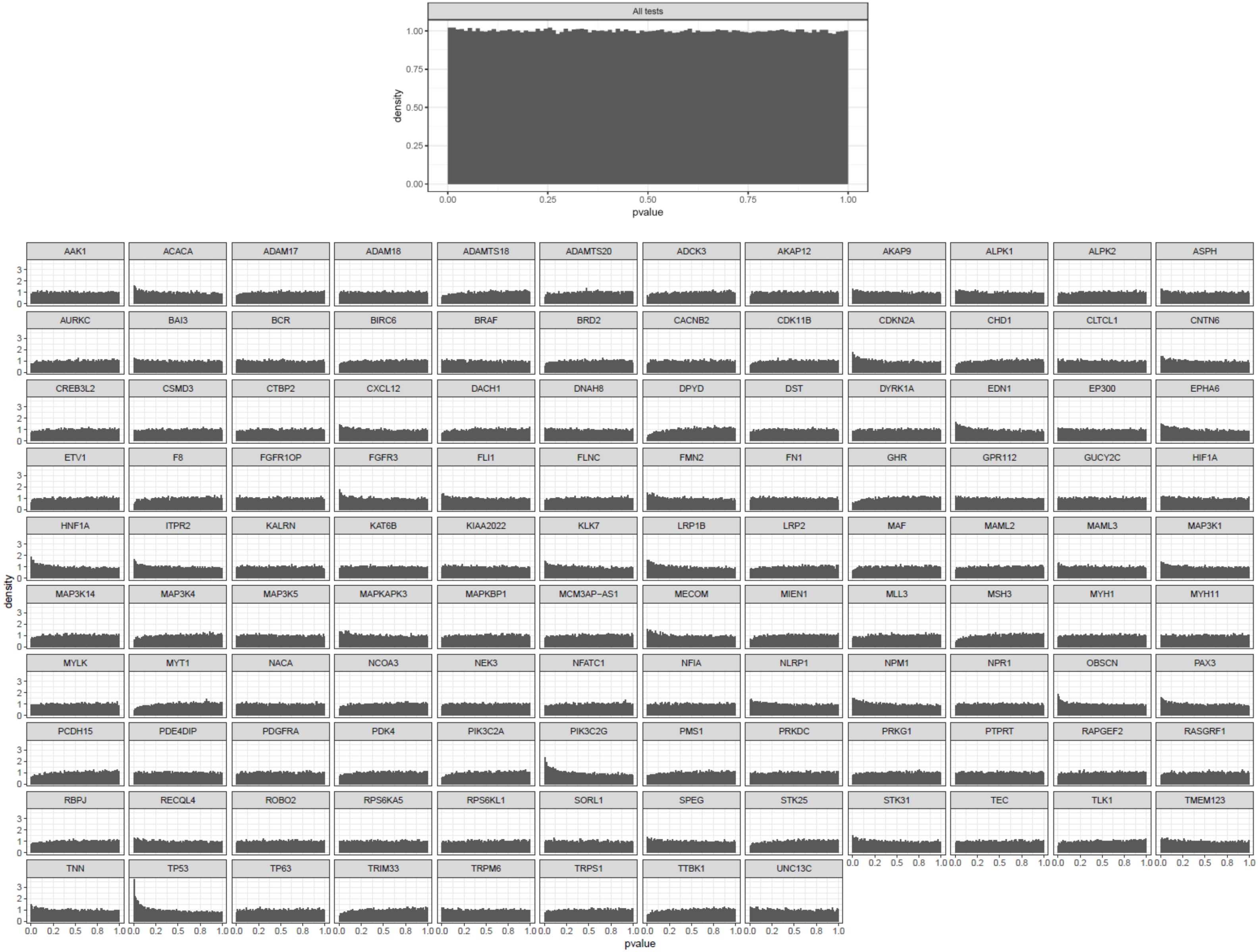

Figure S21. Histogram of P-values of all lethal dependencies in skin carcinoma vs p-values associated with each gene variant.

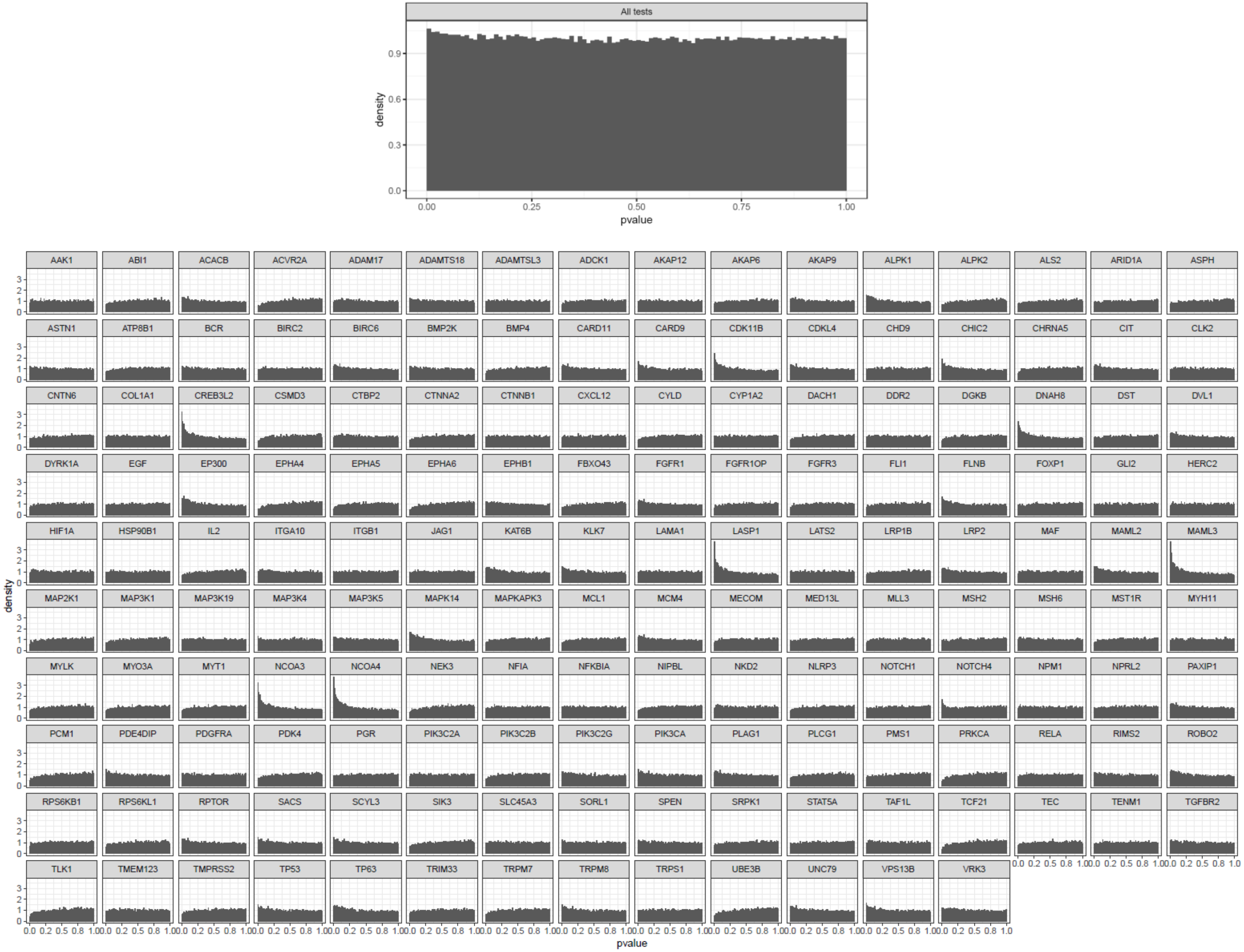

Figure S22. Histogram of P-values of all lethal dependencies in stomach adenocarcinoma vs p-values associated with each gene variant.

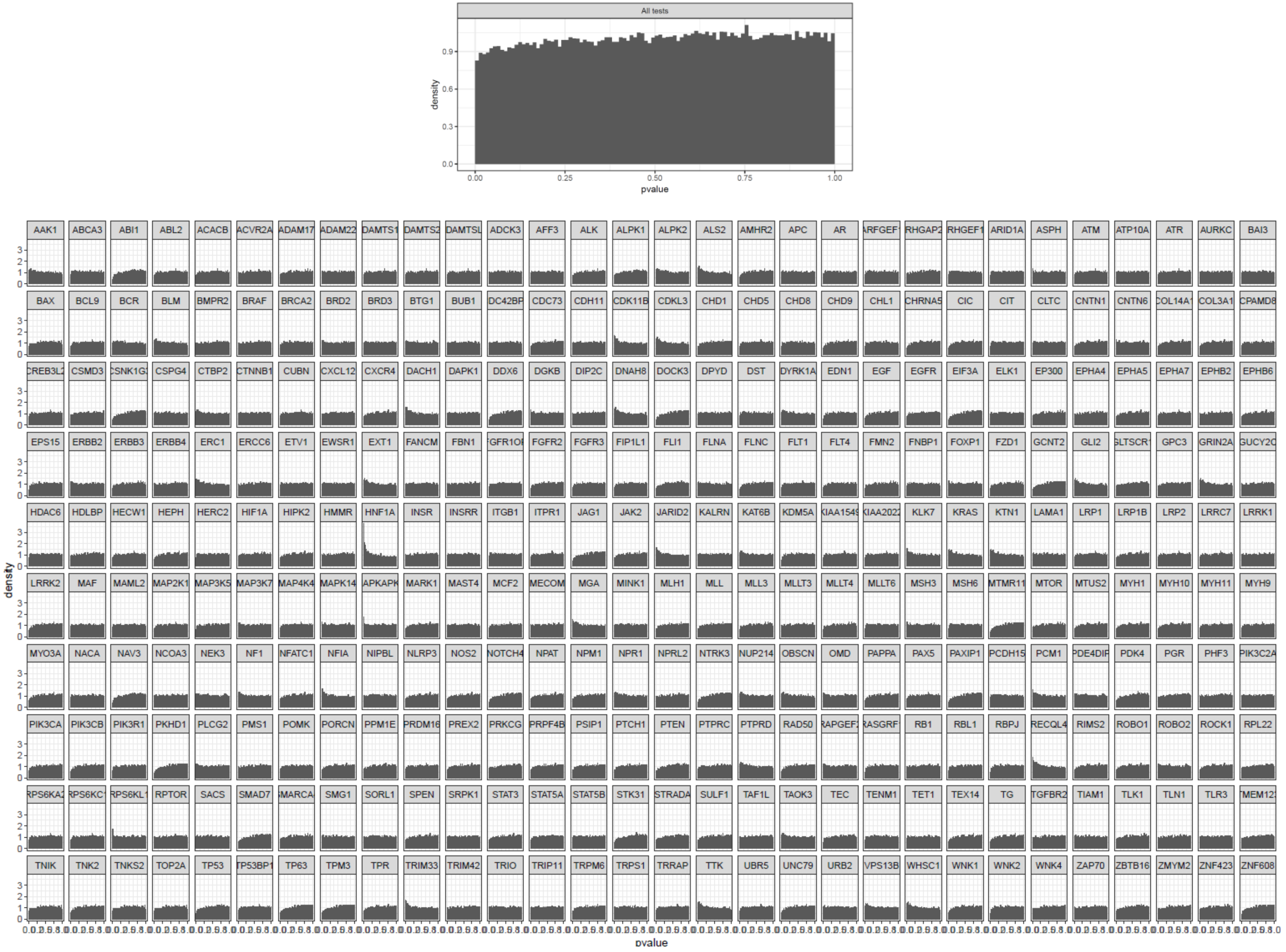

Figure S23. Histogram of P-values of all lethal dependencies in uterine corpus endometrial carcinoma vs p-values associated with each gene variant.

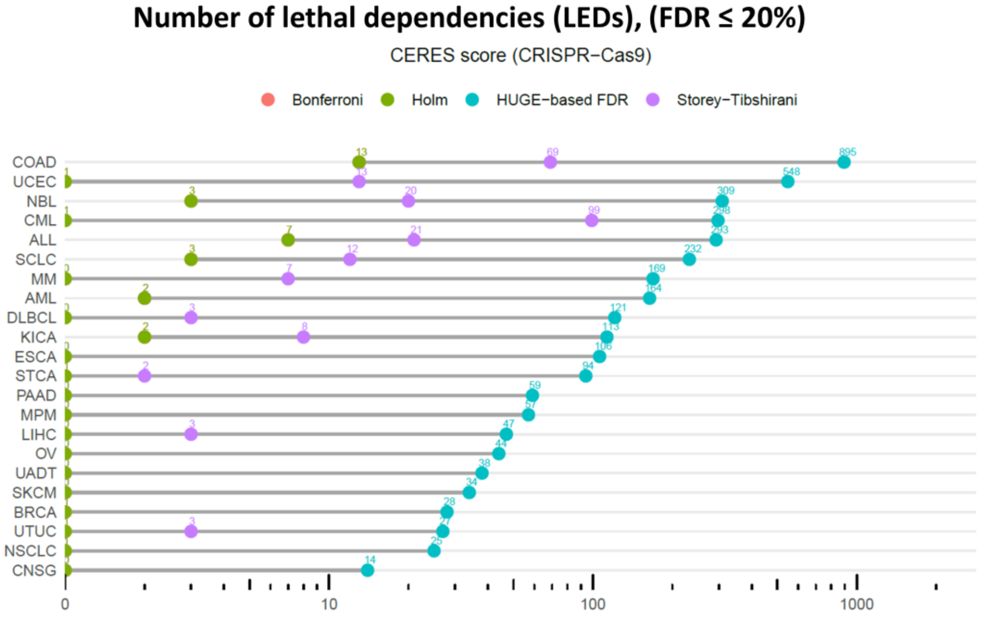

Figure S24. The number of LEDs found (FDR ≤ 20%) in 22 tumors of the CERES score (CRISPR-Cas9) using standard statistical pipelines (Storey-Tibshinary, Bonferroni, and Holm) and the HUGE-based algorithm. Bonferroni and Holm return the same number of hypotheses in all cases. LEGEND: ALL: acute lymphoblastic leukemia; AML: acute myeloid leukemia; BRCA: breast ductal carcinoma; CNSA-IV: central nervous system astrocytoma grade IV; COAD: colon adenocarcinoma; CUADT: upper aero-digestive tract squamous cell carcinoma; DLBCL: diffuse large B-cell lymphoma; ESCA: esophagus squamous cell carcinoma; KIRC: kidney renal clear cell carcinoma; LCC: lung large cell carcinoma; LUAD: lung adenocarcinoma; LUSC: lung squamous cell carcinoma; MM: multiple myeloma; NSCLC: non–small cell lung carcinoma; OS: osteosarcoma; OVAD: ovary adenocarcinoma; PDAC: pancreas ductal carcinoma; SCLC: small cell lung carcinoma; SKCM: skin carcinoma; UCEC: endometrium adenocarcinoma.

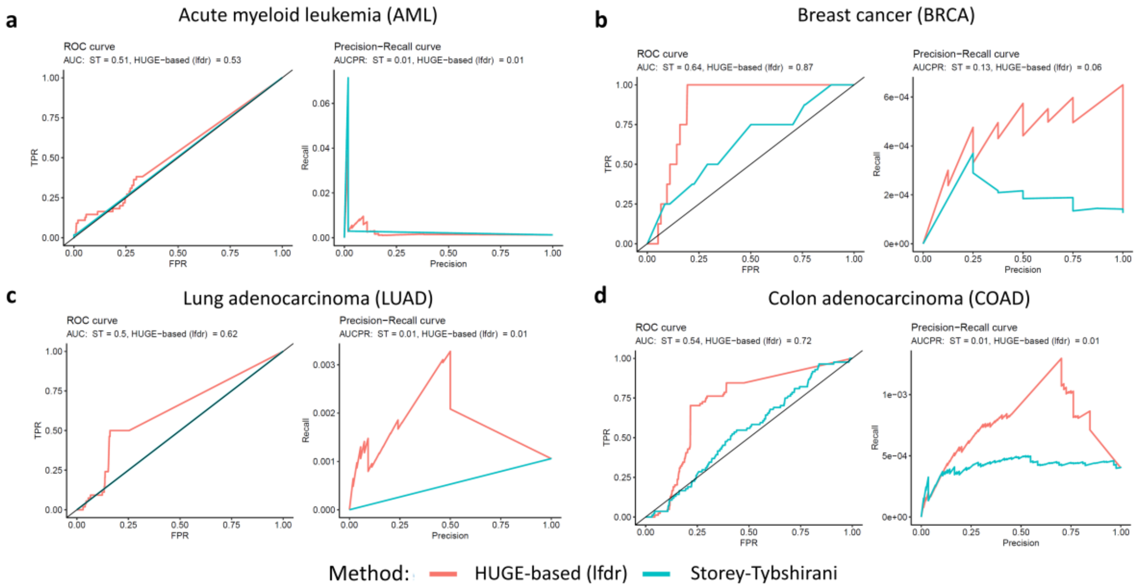

Figure S25. ROC and precision-recall curves of four tumor types. True positives are extracted from the somatic genomic variants in the knowledgebase of the Variant Interpretation for Cancer Consortium (VICC) for four different cancers. Each of the panels show a) AML, b) BRCA, c) LUAD and d) COAD. We selected associations indicated for each tumor type that are within the three highest levels of confidence (Level A: Evidence from professional guidelines or FDA-approved therapies relating to a biomarker and disease; Level B: Evidence from clinical trials or other well-

powered studies in clinical populations, with expert consensus; and Level C: Evidence for therapeutic predictive markers from case studies, or other biomarkers from several small studies, or evidence for biomarker therapeutic predictions for established drugs for different indications). Both the ROC and the Precision Recall curves have larger area for HUGE than fot the ST method.

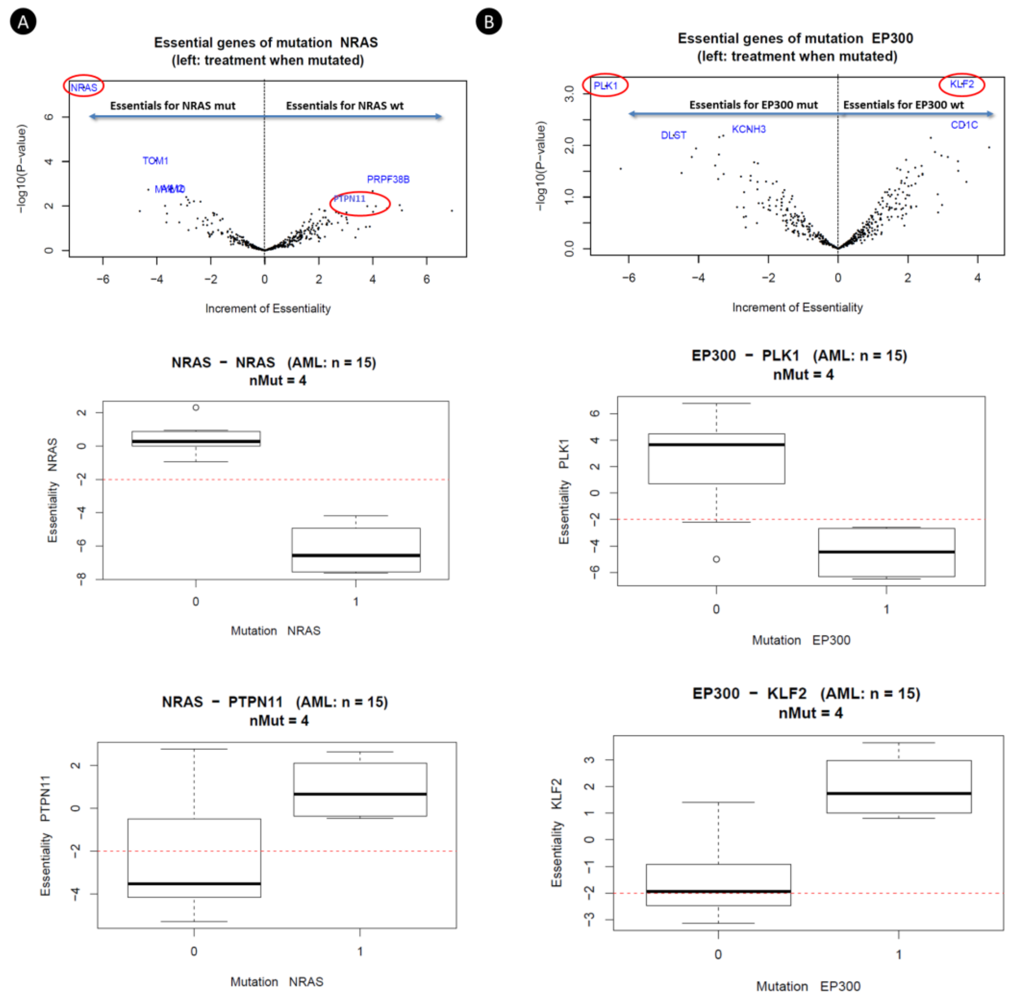

Figure S26. Volcano plot of Synthetic lethal genes related to *NRAS*-mutated (A) and *EP300*-mutated (B) phenotypes. Increment of Essentiality and -log10 (P-value) are shown y x-axis and y-axis respectively. Boxplots of AML P-values of same genes' synthetic lethal: *NRAS, PTPN11, PLK1* and *KLF2.*

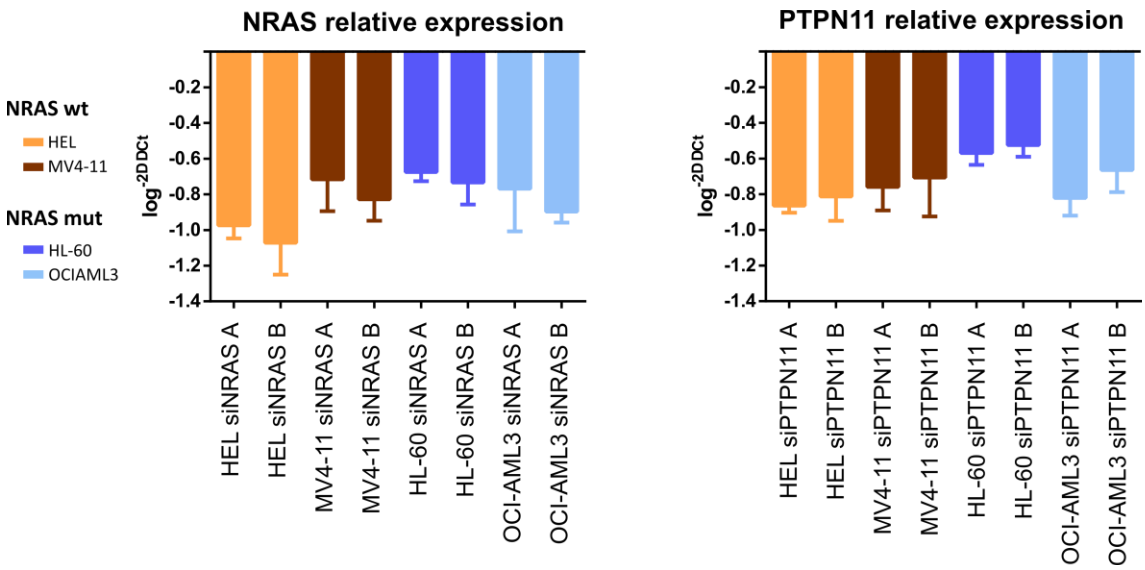

Figure S27. mRNA expression of *NRAS* and *PTPN11* genes after nucleofection with the specific siRNAs. Data are referred to *GUSB* gene and an experimental group nucleofected with negative control siRNA.
