# Supplementary material for: Identifying Lethal Dependencies with HUGE Predictive Power
Source: Cancers (Basel). 2022 Jul 1;14(13):3251. doi: 10.3390/cancers14133251 (PMC9264916; doi:10.3390/cancers14133251)
Supplement: Supplementary file 1 [file cancers-14-03251-s001.zip › 2022_Gimeno, San Jose, et al. Suppl. Methods.pdf]

Supplementary methods

Identifying Lethal Dependencies with HUGE Predictive Power

Marian Gimeno[1,‡], Edurne San José-Enériz[2,3,‡], Angel Rubio[1,4], Leire Garate[3,5], Estíbaliz Miranda[2,3], Carlos Castilla[1], Xabier Agirre[2,3,*], Felipe Prosper[2,3,5,*], and Fernando Carazo[1,4*].

[1]Departamento de Ingeniería Biomédica y Ciencias, TECNUN, Universidad de Navarra, San Sebastian, Spain.

[2]Programa Hemato-Oncología, Centro de Investigación Médica Aplicada, IDISNA, Universidad de Navarra, Pamplona, Spain.

[3]Centro de Investigación Biomédica en Red de Cáncer (CIBERONC), Madrid, Spain.

[4]Instituto de Ciencia de los Datos e Inteligencia Artificial (DATAI), Universidad de Navarra, 31080 Pamplona, Spain

[5]Departamento de Hematología, Clinica Universidad de Navarra, Universidad de Navarra, Pamplona.

‡MG and ESJ-E contributed equally to this work.

*These authors share senior authorship. Correspondence and requests for materials should be addressed to F.P. (email: fprosper@unav.es), F.C. (email: fcarazo@tecnun.es) and X.A. (email: xaguirre@unav.es).

Contents

SUPPLEMENTAL METHODS 3
Section 1. Cell culture 3
Section 2. Cell transfection 3
Section 3. Cell proliferation assay 3
Section 4. Quantitative-PCR (Q-PCR) 3
Section 5. Statistical pipeline 4
Section 6. A larger number of positives outperforms specificity and sensitivity 6
SUPPLEMENTARY REFERENCES 9

SUPPLEMENTAL METHODS

Section 1. Cell culture

The AML cell lines HL-60, HEL, MV4-11 and OCI-AML3 were maintained in culture in RPMI-1640 medium supplemented with 10% fetal bovine serum (Gibco, Grand Island, NY), penicillin/streptomycin (BioWhitaker, Walkersvill, MD) at 37 °C in a humid atmosphere containing 5% CO2. All cell lines were tested for mycoplasma (MycoAlert Sample Kit, Cambrex) and were authenticated by performing a short tandem repeat allele profile.

Section 2. Cell transfection

Cells were passaged 24 hours before nucleofection, and cells for nucleofection were in their logarithmic growth phase. The transfection of siRNAs was done with the Nucleofector II device (Amaxa GmbH, Köln, Germany) following the Amaxa guidelines. Briefly, 1×10^6 of HL-60, HEL, MV4-11 and OCI-AML3 cells were resuspended in 100 µL of supplemented culture medium or solution V in the case of HL-60 cells, with 75nM of NRAS or PTPN11 siRNAs or Silencer Select Negative Control-1 siRNA (Ambion, Austin, TX) and nucleofected with the Amaxa nucleofector apparatus using programs A030 (HEL, MV4-11 and OCI-AML3) or T019 (HL-60). We used two different siRNAs against *NRAS* target (siNRAS A: GAACCACUUUGUAGAUGAA; siNRAS B: AAGGACAGTTGATACAAAA) and *PTPN11* (siPTPN11 A: AGAUGUCAUUGAGCUUAAA; siPTPN11 B: GAAAGAAGCAGAGAAAUUA) to demonstrate that the results obtained with siRNA nucleofection are not due to a combination of inconsistent silencing and sequence specific off-target effects. Silencer Select Negative Control-1 siRNA was used to demonstrate that the nucleofection did not induce non-specific effects on gene expression. Nucleofection was performed twice with a 24 hours interval. 48 h after the second nucleofection, the *NRAS* and *PTPN11* mRNA expression was analyzed by Q-PCR (*GUSB* was employed as the reference gene). Cell proliferation was analyzed 0, 2 ,4 and 6 days after two repetitive transfections. Transfection efficiency was determined by flow cytometry using the BLOCK IT Fluorescent Oligo (Invitrogen Life Technologies, Paisley, UK).

Section 3. Cell proliferation assay

Cell proliferation was analyzed using the CellTiter 96 Aqueous One Solution Cell Proliferation Assay (Promega, Madison, W). This is a colorimetric method for determining the number of viable cells in proliferation. For the assay, 100 µL of nucleofected cells were plated in 96 wells plates 0, 2, 4 and 6 days after the last nucleofection. Plates with suspension cells were centrifuged at 800 g for 10 minutes and medium was removed. Then, cells were incubated with 100 µL/well of medium and 20 µL/well of CellTiter 96 Aqueous One Solution reagent. The plates were incubated for 1-4 hours, depending on the cell line at 37 ºC in a humidified, 5 % CO2 atmosphere. The absorbance was recorded at 490 nm using 96-well plate readers until absorbance of control cells without treatment was around 0.8. The background absorbance was measured in wells with only cell line medium and solution reagent. First, the average of the absorbance from the control wells was subtracted from all other absorbance values. Data were calculated as the percentage of total absorbance of siRNA transfected cells/absorbance of control cells.

Section 4. Quantitative-PCR (Q-PCR)

The expression of *NRAS* and *PTPN11* was analyzed by Q-PCR in HL-60, HEL, MV4-11 and OCI-AML3 AML cell lines. First, total mRNA was extracted with Trizol© Reagent 5791 (Life

Technologies, Carlsbad, CA, USA) following the manufacturer instructions. RNA concentration was quantified using NanoDrop Specthophotometer (NanoDrop Technologies, USA). cDNA was synthesized from 1 µg of total RNA using the PrimeScript RT reagent kit (Perfect Real Time) (Cat No RR037A, TaKaRa) following the manufacturer's instructions. The quality of cDNA was checked by a multiplex PCR that amplifies *PBGD*, *ABL*, *BCR* and *β2-MG* genes. Q-PCR was performed in a QuantStudio 5 Real-Time PCR System (Applied Biosystems), using 20 ng of cDNA in 2 µL, 1 µL of each primer at 5µM (NRAS F: 5'-CGCACTGACAATCCAGCTAA-3'; NRAS R: 5'-CCAACAAACAGGTTTCACCA-3'; PTPN11 F: 5'-CGGAGCCTGAGCAAGGAG-3'; PTPN11 R: 5'-CTGCCTCCACACCAGTGATA-3'; GUSB F:5' gaaaatatgtggttggagagctcatt-3'; GUSB R: 5'-ccgagtgaagatccccttttta-3'), 6 µL of SYBR Green PCR Master Mix 2X (Cat No 4334973, Applied Biosystems) in 12 µL reaction volume. The following program conditions were applied for Q-RT-PCR running: 50 ºC for 2 min, 95 ºC for 60 s following by 45 cycles at 95 °C for 15 s and 60 °C for 60 s; melting program, one cycle at 95 °C for 15 s, 40 °C for 60 s and 95 °C for 15 s. The relative expression of each gene was quantified by the Log $2^{(-\Delta\Delta Ct)}$ method using the gene *GUSB* as an endogenous control.

Section 5. Statistical pipeline

A statistical pipeline (**Figure S1**) has been developed to solve the problem of identifying subrogate mutation biomarkers of gene essentiality (RNAi target genes).

Data of RNAi libraries (more than 17,000 knocked-down genes in 412 cancer cell lines) of the project Achilles [1] were integrated with their corresponding mutational profiles (mutations in ~1600 genes; **Figure 1A**) obtained from the Cancer Cell Line Encyclopedia (CCLE)[2] and Shao et al. [3]. We filter out the mutations that meet any of the following criteria: (i) common polymorphisms, (ii) allelic fraction < 10%, (iii) putative neutral variants (missenses present in less than 2 warm-blooded vertebrates), or (iv) located outside of the coding sequence. We used the DEMETER score [4,5] as a measure of gene essentiality of the RNAi libraries of the project Achilles [1]. DEMETER quantizes the competitive proliferation of the cell lines controlling the effect of off-target hybridizations of siRNAs by solving a complex optimization problem. The more negative the DEMETER score is, the more essential the gene is for a cell line. We imputed missing elements of DEMETER using the nearest neighbor averaging algorithm [6]. In addition, we collected gene expression patterns from RNA-seq data [7] to confirm that essential genes are expressed when they are essential.

Based on DEMETER data, we first identified genes that were essential for a selected tumor subtype. Essential genes were required to meet several criteria: i) they must be essential for at least 20% samples of the selected cancer subtype, ii) they must be specific to the cancer type under study, i.e. they must be non-essential for other cancer types and iii) they must be expressed before RNAi experiment (>1TPM at least in 75% samples). These filters reduce the number of hypotheses in the statistical analysis.

We developed a statistical algorithm to identify genes whose essentiality is highly associated with the mutational status of other genes. Dealing with this statistical issue implies solving a large multiple hypotheses problem (more than one million hypotheses). In similar scenarios, traditional corrections -such as Benjamini-Hochberg (BH), Bonferroni or Holm- showed very few or no gene-biomarker pairs for a given FDR [8]. In order to overcome this problem, we developed a covariate-based statistical approach -similar to the Independent Hypothesis Weighting procedure [8].

Statistical model: Let e denote the number of RNAi target genes and n denote the number of screened samples. Let **D** be an $e \times n$ matrix of essentiality whose entries d_{ij} represent the

DEMETER score for the RNAi target *i* in sample *j*. Let **m** be a $m \times n$ dichotomized matrix whose entry m_{ij} denotes whether sample *j* is mutant or not according the previous criteria:

$$m_{ij} = \begin{cases} 1, & if mutant(MUT) \\ 0, & if\ wild - type\ (\mathrm{WT}) \end{cases}, \qquad (1)$$

Let **s** be a subset of n' cell lines that yields an essentiality vector$\boldsymbol{d_s} = \left(d_{e_{s_1}}, \ldots, d_{e_{s_{n'}}}\right)$ for the eth RNAi target. Let $\boldsymbol{m_s} = \left(m_{s_1} \ldots, m_{s_n}\right)$ be the expression vector of a putative gene biomarker. The null hypotheses are defined as:

$$H_0^g: E(\boldsymbol{d_s} | \boldsymbol{m_s} \in MUT) = E(\boldsymbol{d_s} | \boldsymbol{m_s} \in WT) \qquad (2)$$

This null hypothesis is therefore: "the expected essentiality of a gene knock-down is identical in mutant and wild-type cell lines". To test this hypothesis, we used a moderated t-test implemented in *limma*[9]. We applied this test for each RNAi target and all the mutations to get the corresponding p-values. Dealing with these p-values implies correcting for multiple hypotheses.

In order to face these challenges, we followed a methodology similar to the IHW (Independent Hypothesis Weighting) procedure [8], which increases the power of a test by grouping the results using covariates. We show in the main manuscript that the number of positives returned by IHW is larger for all the cancer datasets and therefore, this method outperforms the standard FDR estimation both in sensitivity and specificity (as shown in the lemma of the following section in the supplementary material).

In our case, we divided the p-values corresponding to all the tests into 2n groups, where n is the number of knock-down genes.

For each of these groups, we computed the local false discovery rate (local fdr) [10]. The local fdr estimates, for each test, the probability of the null hypothesis to be true, conditioned on the observed p-values. The formula of the local fdr is the following:

$$P(H_0 | z) = localfdr(z) = \frac{\pi_0 f_0(z)}{f(z)}, \qquad (3)$$

where *z* are the observed p-values, π_0 is the proportion of true null hypotheses –estimated from the data-, $f_0(z)$ the empirical null distribution –usually a uniform (0,1) distribution for well-designed tests- and $f(z)$ the mixture of the densities of the null and alternative hypothesis, also estimated from the data.

As stated in [47], "the advantage of the local fdr is its specificity: it provides a measure of belief in gene i's 'significance' that depends on its p-value, not on its inclusion in a larger set of possible values" as it occurs, for example with q-values or the standard False Discovery Rate. The local fdr and π_0 were estimated using the Bioconductor's R Package *qvalue* [11].

For a selected cohort of cells, the algorithm outputs a ranking of significant gene pairs (GPs) that consist of a couple of genes in which the first one is essential depending on the mutational status of the other. For the final ranking, we selected those GPs that showed a p-value < 0.05 and local FDR ≤ 0.6, |ΔDEMETER| > 2. Additionally, we interrogated which of these pairs had direct relationships (co-expressed, annotated in the same pathway database or contained in a common experiment) in the STRING database [12] to ensure there is an established biological relationship between the essential gene and the subrogate biomarker. This biological double-check is not necessary and can be omitted when the researcher looks for novel relationships.

Section 6. A larger number of positives outperforms specificity and sensitivity

Lemma: *Let us consider two methods that correct multiple hypothesis test, and let us consider that both methods provide a different number of positives for the same FDR. Then, the method that provides a larger number of positives has more statistical power. It is also more specific and sensitive.*

The power or sensitivity of a statistical test is the probability that the test correctly rejects the null hypothesis H_0 when the alternative hypothesis H_1 is true. Its value is *TP/(TP+FN)*.

Let's consider that the estimation of the FDR is performed by two tests A and B and both tests have the same False Discovery Rate (20% for example). The FDR will be

$$FDR = \frac{FP_A}{TP_A + FP_A} = 1 - \frac{TP_A}{TP_A + FP_A} = \frac{FP_B}{TP_B + FP_B} = 1 - \frac{TP_B}{TP_B + FP_B} (1a)$$

The power of each test will be

$$PW_A = 1 - \beta_A = \frac{TP_A}{TP_A + FN_A} (1b)$$

$$PW_B = 1 - \beta_B = \frac{TP_B}{TP_B + FN_B} (1c)$$

Since both tests are performed on the same dataset, the number of true null hypothesis H_0 (FP + TN) and true alternative hypothesis H_1 (TP+FN) will be identical, i.e.,

$$FP_A + TN_A = FP_B + TN_B (1d)$$

$$TP_A + FN_A = TP_B + FN_B (1e)$$

Notice that the denominators of the expression of the power (eq (1b) and (1c)) are identical according to (1e).

The total number of positives returned by each test is $TP_A + FP_A$ and $TP_B + FP_B$. Let's assume that method A, returns more positives than method B, i.e.

$$TP_A + FP_A > TP_B + FP_B (2)$$

Using eq. (1a), and (2)

$$TP_A = (1 - FDR)(TP_A + FP_A)(3a)$$

And,

$$TP_B = (1 - FDR)(TP_B + FP_B)(3b)$$

Since (2), the righthand member of equation (3a) is larger than the righthand member of equation (3b) and therefore,

$$TP_A > TP_B (4)$$

As a result,

$$PW_A > PW_B \blacksquare$$

Corolary I. Since $PW_A = 1 - \beta_A$ the type II error using A is smaller than using B.

$$\beta_A < \beta_B$$

Corolary II. The type I error is

$$\alpha_A = \frac{FP_A}{FP_A + TN_A}$$

And the sensitivity is:

$$1 - \alpha_A = \frac{TN_A}{FP_A + TN_A}$$

By (1e) and (4), it is straightforward to conclude that

$$\alpha_A < \alpha_B$$

Therefore, the method that provides a larger number of positives outperforms the other both in terms of specificity and sensitivity (or type I and type II errors).

SUPPLEMENTARY REFERENCES

1. Cowley GS, Weir BA, Vazquez F, et al. Parallel genome-scale loss of function screens in 216 cancer cell lines for the identification of context-specific genetic dependencies. *Sci. data*. 2014;1:140035.

2. Barretina J, Caponigro G, Stransky N, et al. The Cancer Cell Line Encyclopedia enables predictive modelling of anticancer drug sensitivity Supp. *Nature*. 2012;483(7391):603–7.

3. Shao DD, Tsherniak A, Gopal S, et al. ATARiS: Computational quantification of gene suppression phenotypes from multisample RNAi screens. *Genome Res.* 2013;23(4):665–678.

4. McFarland JM, Ho Z V., Kugener G, et al. Improved estimation of cancer dependencies from large-scale RNAi screens using model-based normalization and data integration. *Nat. Commun.* 2018;9(1):1–13.

5. Tsherniak A, Vazquez F, Montgomery PG, et al. Defining a Cancer Dependency Map. *Cell*. 2017;170(3):564-576.e16.

6. Brown P, Hastie T, Tibshirani R, Botstein D, Altman RB. Missing value estimation methods for DNA microarrays. *Bioinformatics*. 2001;17(6):520–525.

7. Tatlow PJ, Piccolo SR. A cloud-based workflow to quantify transcript-expression levels in public cancer compendia. *Sci. Rep.* 2016;6:39259.

8. Ignatiadis N, Klaus B, Zaugg JB, Huber W. Data-driven hypothesis weighting increases detection power in genome-scale multiple testing. *Nat. Methods*. 2016;13(7):577–580.

9. Ritchie ME, Phipson B, Wu D, et al. Limma powers differential expression analyses for RNA-sequencing and microarray studies. *Nucleic Acids Res.* 2015;43(7):e47.

10. Efron B, Tibshirani R. Empirical Bayes methods and false discovery rates for microarrays. *Genet. Epidemiol.* 2002;23(March):70–86.

11. Storey JD. A direct approach to false discovery rates. *J. R. Stat. Soc. Ser. B Stat. Methodol.* 2002;64(3):479–498.

12. Szklarczyk D, Gable AL, Lyon D, et al. STRING v11: Protein-protein association networks with increased coverage, supporting functional discovery in genome-wide experimental datasets. *Nucleic Acids Res.* 2019;47(D1):D607–D613.

13. Romero JP, Ortiz-Estévez M, Muniategui A, et al. Comparison of RNA-seq and microarray platforms for splice event detection using a cross-platform algorithm. *BMC Genomics*. 2018;
